# The Driver Role of Pathologists in Endocrine Oncology: What Clinicians Seek in Pathology Reports

**DOI:** 10.1007/s12022-023-09768-y

**Published:** 2023-05-11

**Authors:** Shereen Ezzat, Wouter W. de Herder, Marco Volante, Ashley Grossman

**Affiliations:** 1 Endocrine Oncology Site Group, Princess Margaret Cancer Centre, University of Toronto, Toronto, ON Canada; 2 Department of Internal Medicine, Sector of Endocrinology, Erasmus MC Cancer Institute, Erasmus University Medical Center, Rotterdam, The Netherlands; 3 Department of Oncology, University of Turin, San Luigi Hospital, Regione Gonzole 10, 10043 Orbassano, Turin, Italy; 4 Barts and the London School of Medicine, University of London, London, UK; 5 Green Templeton College, University of Oxford, Oxford, UK

**Keywords:** Endocrine tumors, Pathology reporting, Imaging, Biochemical testing

## Abstract

Endocrine neoplasia represents an increasingly broad spectrum of disorders. Endocrine neoplasms range from incidental findings to potentially lethal malignancies. In this paper, we cover the impact of pathology in the interpretation of the clinic-pathological, genetic, and radiographic features underpinning these neoplasms. We highlight the critical role of multidisciplinary interactions in structuring a rational diagnostic and efficient therapeutic plan and emphasize the role of histopathological input in decision-making. In this context, standardized pathology reporting and second opinion endocrine pathology review represent relevant tools to improve the overall diagnostic workup of patients affected by endocrine tumors in every specific scenario. In fact, although a relevant proportion of cases may be correctly identified based on clinical presentation and biochemical/imaging investigations, a subset of cases presents with atypical findings that may lead to an inappropriate diagnosis and treatment plan based on a wrong pathological diagnosis if all pieces of the puzzle are not correctly considered. Pathologists have a responsibility to actively guide clinicians before and during surgical procedures to prevent unnecessary interventions. In all areas of endocrine pathology, pathologists must understand the complexity of tissue preservation and assay sensitivities and specificities to ensure the optimal quality and interpretation of diagnostic material. Finally, pathologists are central actors in tumor tissue biobanking, which is an expanding field in oncology that should be promoted while adhering to strict ethical and methodological standards.

## General Overview

The basic clinical approach to patients with diseases of the endocrine system is the classic one of “history, examination, and investigation.” In particular, obtaining a full, comprehensive, and detailed history is critical. In contrast to other organ systems, such as in cardiology and gastroenterology, where the major symptom complexes usually relate to the specific system, such as chest pain in cardiology or diarrhea in gastroenterology, the effect of endocrine disorders can be widespread, often rather disparate, and difficult to separate from other “functional” disorders. In many cases, the patient may present to the specialist endocrinologist with a suspicion of a disorder of one or other of the classic “glands,” and in such cases, following through the history may be relatively straightforward, with highly directed questions and a focused interrogation. But in many other situations, the presenting symptoms may appear vague and unconnected, and then the history, the structured questions including past medical history, family and social history, may all be critical.

In some instances, and not infrequently, the patient may present to the endocrine consultant after having had a journey of years of “random” encounters. These may include a number of disconnected diagnostic and therapeutic procedures, which, at the time of initial presentation, were not considered as a whole. The patient may have become frustrated and discouraged, feeling that none of their consultant specialists has fully addressed their concerns.

Examination of all patients needs to be comprehensive, as localizing features for many endocrine tumors may be disparate and apparently unrelated at first sight. On the other hand, the typical appearance of the patient with acromegaly, Cushing’s syndrome, or a carcinoid flush may be readily apparent as the patient enters the consulting room.

It has been said that 70% of diagnoses are made after a thorough history, a further 10% concluded after the physical examination, while only a small percentage are established, as opposed to confirmed, after relevant investigation: for the remainder, a certain organic diagnosis may remain elusive [1].

Thus, the investigations should follow the suspected differential diagnosis/diagnoses, to confirm or exclude them; there may be a screen of “routine” blood tests, but in endocrine investigation, all test procedures should be aimed to offer a binary distinction between the presence or absence of an endocrine disorder, specifically to establish the presence of an endocrine tumor, or at least suggest relative probabilities. This will usually take the form of biochemical assessments of a body fluid, usually blood but including urine and saliva, followed by appropriate imaging techniques, anatomical or functional, or both. It is important that in most cases the imaging is led by the biochemistry, and not the other way round, except in the cases of “incidentalomas,” although these are an increasing problem. One should certainly be careful to avoid a “let’s test everything” strategy, as one may end up with more rather than less uncertainty.

Finally, when the history and physical examination have suggested a diagnosis of a possible endocrine tumor, and this is confirmed by biochemical investigation and localized by appropriate imaging, the judgement call of the optimal management plan will often depend on establishing the precise pathology to lead to the most appropriate therapy—surgical, medical, or possibly simple surveillance. The histopathological input is usually highly influential in assisting in this decision, although in many instances the results of imaging will also help in determining the necessity for such pathology. However, if the surgical route is taken, then a post-operative histopathological review is essential in establishing the precision (or otherwise) of the original diagnosis and the subsequent course of action. As clinicians, we generally consider the histopathological analysis to be the “gold standard” in establishing a unifying “diagnosis.” While it will often be concordant with pre-biopsy or surgical diagnosis, in many cases, only a firm and complete histological analysis will provide the necessary information for the optimal prognostic and therapeutic plan.

All of this information—clinical findings, biochemical, imaging, and histopathological data—are often best discussed at a multidisciplinary team (MDT) meeting, or a “tumor board,” to inform decision-making. However, it is usually the case that only the specialist endocrinologist in charge of the case is aware of all the background and social details, such that the MDT can only *assist* in decision-making rather than *mandating* a course of action. But in every case, the histopathology, increasingly with precision immunohistochemistry and molecular analyses, will be vital in optimizing a course of action for the patient. Not infrequently, an initial histopathological diagnosis by a non-specialist pathologist will need to be revisited in light of other information. A pituitary specimen may not have been subject to detailed immunocytochemistry, or (unfortunately, not rarely) a patient will have been diagnosed and treated for a pancreatic adenocarcinoma when the true diagnosis may be a low-grade well-differentiated neuroendocrine tumor. This emphasizes the need for specialist histopathologists and, additionally, the recognition of such uncertainty by non-specialist pathologists.

We will now show how this clinical approach applies to patients with endocrine tumors of various types and specifically how the input of the pathologist influences our decision-making in different clinical settings.

## Standardized Pathology Reporting: from Descriptive to Informative Pathology Reports

The key step in providing patients with a management plan is the establishment of solid histopathologic findings. Absent information should not be misinterpreted as a negative finding. Instead, the presence or absence of specific comments provides a line of communication to assure clinicians that their most relevant questions are being addressed. The use of standardized reporting formats should help obviate many of the more egregious errors.

Over the last 20 years, there has been a paradigm shift in cancer pathology reporting, evolving from narrative records of pathology findings to structured reports detailing parameters that represent the main diagnostic, prognostic, and predictive elements. This evolution is the consequence of the increasing complexity of knowledge in cancer biology and therapy. Datasets for pathological reporting are critical not only for individual patient care but also for cancer registries, clinical trials, epidemiology research, resource planning, and quality indicator programs. In addition, they can be used as educational tools for pathologists-in-training or in developing countries providing cost-effective means of achieving an international standard of pathology reporting. They embrace the main goals of pathology reporting: (i) the use of appropriate and intelligible terminology, (ii) the use of uniformly accepted rules (classifications), (iii) the need to formulate timely and informative diagnoses, and (iv) the need to adhere to quality control programs. Dataset standards have been traditionally set by national colleges of pathologists, such as those in the USA (College of American Pathologists. Cancer protocols and checklists. 2021. Available at: https://www.cap.org/protocols-and-guidelines/cancer-reporting-tools/cancer-protocol-templates) or in the UK (Royal College of Pathologists, UK. Cancer datasets and tissue pathways. 2021. Available at: https://www.rcpath.org/profession/publications/cancer-datasets.html). However, there are variations between the different datasets and reflected biases related to various aspects of the different pathology settings (from geographical differences in epidemiology to nation-specific health systems). A project aimed at the definition of *universal* schemes for reporting has been established by the *International Collaboration on Cancer Reporting (ICCR) consortium* starting from 2011 [2], by using panels of international content experts and employing a rigorous evidence-based approach. Since then, the ICCR has published more than 50 cancer datasets on its website (International Collaboration on Cancer Reporting. Histopathology reporting guides for cancer specimens. 2013–2021. Available at: http://www.iccr-cancer.org/datasets), and new datasets are under construction. Each dataset includes the internationally agreed elements and useful commentary to guide the reporting pathologist. In endocrine pathology, datasets have been developed for thyroid [3], parathyroid [4], adrenal cortex [5], and paragangliomas [6]. The College of American Pathologists has for many years had synoptic reports for neuroendocrine tumors throughout the gastroenteropancreatic (GEP) system (https://www.cap.org/protocols-and-guidelines/cancer-reporting-tools/cancer-protocol-templates), and the *European Neuroendocrine Tumor Society* (ENETS) recently developed a scheme for synoptic reporting in neuroendocrine neoplasms (NEN) [7]. There has also been a proposal for a synoptic report for pituitary neuroendocrine tumors (PitNETs) [8]. All of these follow a homogeneous scheme with core and non-core elements and adhere to the most recent classification schemes promoted by the WHO [9–12]. Moreover, they help to fit each morphological parameter into the appropriate context, taking into account that every single histological parameter may possess a different impact and may be interpreted differently in various diagnostic scenarios.

For cytology diagnosis in endocrine tumors, standardized reporting is currently only coded for thyroid fine needle aspiration biopsies (FNAB). Several national or international schemes are proposed. The *Bethesda System for Reporting Thyroid Cytopathology* (TBSRTC) is the most widely employed worldwide [13]. Regardless of the scheme employed, it is mandatory that thyroid cytological diagnoses are coded according to the classes included in the classification scheme employed, avoiding any descriptive-only diagnosis.

## Second Opinion Endocrine Pathology Review: A Tool for Improvement

The use of a “second opinion” pathology review is a common but potentially controversial practice. Especially in the era of personalized medicine, accurate pathological diagnosis represents the most important first (and possibly final) step towards treatment. Second opinion in pathology is used as a tool to improve diagnostic accuracy as well as a test for quality control. Some years ago, the *American Society of Clinical Pathology* recommended the review of “second opinion” pathology cases as a tool for error reduction [14]. Moreover, the *Association of Directors of Anatomic and Surgical Pathology* recommended in previous years in-house review of all pertinent pathology slides when a patient is referred to or seeks a clinical opinion at a second institution [15].

A second opinion review may be initiated by pathologists, clinicians, or patients. In the case of pathologists, this approach usually reflects a situation related to the rarity of the disease under evaluation and responds to the need to refer to a pathologist with more experience in the field, although these may be few and far between. This situation particularly applies to endocrine neoplasms, due to their relative rarity in incidence (with special reference to neuroendocrine and adrenal tumors). Clinicians usually ask for a second opinion whenever the first pathology diagnosis does not appear to be compatible with the clinical scenario. In this setting, also, endocrine tumors are frequently heterogeneous in their clinical presentation and biological behavior. Moreover, endocrine manifestations, if present, may not match with the pathology descriptors (including immunophenotyping) and with the final diagnosis in the pathology report. In addition, in several situations, a second opinion pathology review is asked not only to confirm a diagnosis but also to add levels of information that are missing in the first report. However, key to this is the acceptance and realization by the non-specialist pathologist that a second opinion is necessary and does not reflect on their own competence. As already stated before, standardized pathology reporting is a key tool to overcome this issue. On the other side, pathology review may incur undesired additional costs to patients but may prevent unnecessary costs (and toxicities) related to inappropriate treatments [16].

In a recent report, the outcomes of 3738 consecutive second opinion surgical pathology cases from 230 institutions (31 states in the USA and six other countries) were analyzed [17]. Among all cases, 95.5% showed no major discordance, 3.7% had a major discordance with no change in management, while 0.7% had a major discordance implicating a change in management. However, patients affected by endocrine pathologies had the highest rates of major discordance (11.5%), with up to 15.3% discordant cases in patients undergoing thyroid FNAB. In a study reviewing outcomes of a second opinion in the field of adrenocortical tumors, cases with major disagreement that significantly modified the clinical management of patients represented 9% of the total [18]. Moreover, more than 50% of cases were referred for a second opinion because of the absence of relevant information (e.g., the lack of a Ki-67 proliferation index). In NENs of the gastroenteropancreatic (GEP) tract, second opinion pathology review in expert centers for neuroendocrine neoplasms was shown to significantly impact the management of 36% of patients, leading to a new therapeutic indication in 26% [19]. It is worth noting, however, that major discrepancies between first and second opinion pathology diagnoses may be related to poor reproducibility of diagnostic parameters. For example, lung neuroendocrine tumors (NET) subtyping is affected by a very low interobserver agreement (*κ* = 0.32), mostly due to the subjectivity of mitotic index evaluation [20]. Both mitotic index evaluation and Ki67 immunohistochemical assessment are affected by pre-analytical and analytical variables that may have an impact on the clinical decision processes. These include poor fixation, inappropriate duration of fixation, choice of tissue for analysis, the use of different reagents and pretreatments (for Ki-67), and variability of areas under evaluation since a high-power field varies with the microscope, the eyepiece, and the objective lenses used [21, 22]. It may also relate to uncertainties in Ki-67 scoring, the use of “hot spots,” and variations in personal versus computerized reporting schemes [23].

## Clinical Impact of Pathological Findings

Pathology reporting provides the basis for nearly all diagnostic, management, and surveillance plans in endocrine oncology. In the following sections, we will provide a few representative specific examples of where and how critical pathology information drives clinical decision-making as illustrations of the types of problems that may be encountered generally.

### Pathological and Biochemical Correlates

In many cases, the histopathological data complement the clinical and biochemical findings and serve to support the original clinical and therapeutic plan. This is an essential part of the clinical process. However, in other instances, the pathology data may alter or even reverse the initial clinical diagnosis, prognosis, and therapy. As outlined earlier, the traditional course of investigations typically flows with biochemical testing preceding any cytological or histopathological examination. However, there are instances where this sequence is disrupted.

We provide below clinical scenarios to illustrate how biochemical studies are interconnected with histopathologic findings at each of the major body sites.

#### Pituitary Tumors

A 53-year-old female with severe headache, obesity, and type 2 diabetes mellitus is diagnosed with a pituitary tumor that is considered to be non-functioning; however, following pituitary surgery, the tumor stains weakly for adrenocorticotrophin (ACTH), is TPIT positive, and has a Ki-67 of 4%. This leads to a revised diagnosis of a silent or “whispering” corticotroph pituitary neuroendocrine tumor, intensification of follow-up, and a lowered threshold for further interventions.

A 55-year-old male presents with visual loss, hypogonadism, and a serum prolactin of 1200 mU/L (~ 60 ng/ml). He is given a trial of dopamine agonists but is unable to tolerate these and is subjected to trans-sphenoidal pituitary surgery: in this case, the tumor is immunonegative for PIT1 and prolactin, but stains for SF1, GATA3, and focally for LH, and the diagnosis is changed to “gonadotroph tumor.” He had disconnection hyperprolactinemia, and this necessitates a different follow-up strategy.

A 64-year-old female is referred by her optometrist to an endocrinologist with a report of failing vision and a unilateral visual field defect; a large pituitary mass is identified. Detailed investigation demonstrates mild diabetes insipidus (arginine vasopressin deficiency (AVP-D) among other defects, but a more detailed history reveals that she had breast cancer treated 10 years previously. A pathology examination of the resected pituitary lesion confirms a breast cancer metastasis, and she is referred for further chemotherapy.

**Fig. 1:**
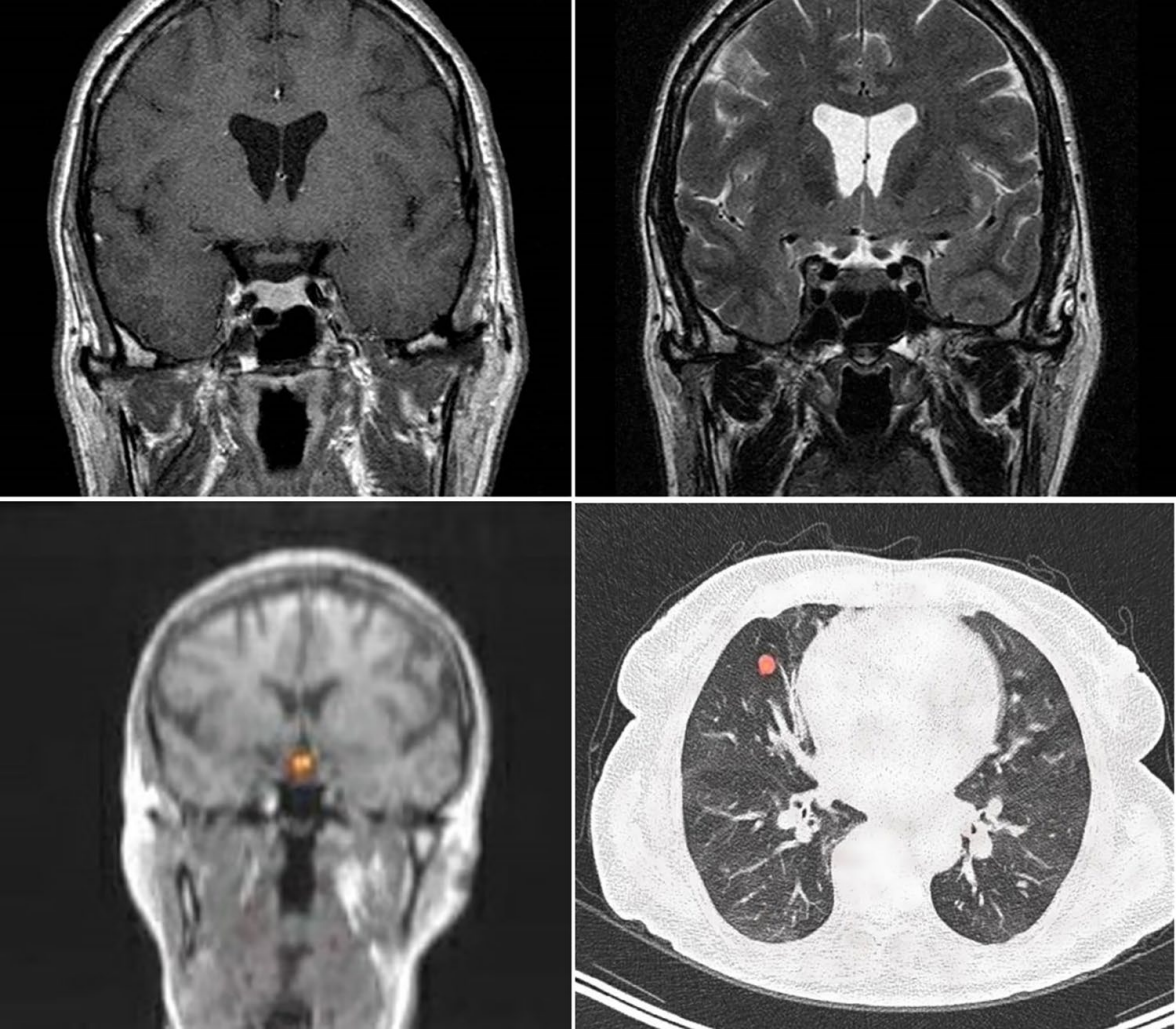
Ectopic acromegaly. 73-year-old woman with acromegaly; serum IGF-1 levels were fivefold x upper limits of normal (ULN), and GHRH levels were threefold ULN. Upper panels, from left to right: T1 weighted + Gadolinium-diethylenetriamine-pentaacetic acid (Gd-DTPA) MRI shows a slight diffuse enlargement of the pituitary; T2W MRI shows a hypointense signal in the pituitary. Lower panels, from left to right: ^68^Ga-DOTATAE PET MRI shows uptake in the pituitary area; ^68^Ga-DOTATE PET CT shows uptake in an SSTR-positive bronchial NET in the right middle lobe. Final diagnosis was bronchial NET with ectopic GHRH production [77]

Finally, in a classic case, a 54-year-old female presents with typical history, examination, and investigation confirming acromegaly, but histopathology of the tumor post-surgery shows retention of the reticulin pattern compatible with hyperplasia rather than a primary pituitary neoplasm. This leads to a search for a source of ectopic growth hormone-releasing hormone (GHRH), confirmed by a grossly elevated circulating GHRH blood level and the presence of a pancreatic mass indicative of a neuroendocrine tumor secreting GHRH ectopically [24]. Another example of ectopic GHRH production is illustrated in Fig. 1.

In some instances, the detection of new histopathologic or unexpected findings may trigger new biochemical and radiological investigations, as was the case with the patient above with somatotroph hyperplasia.

#### Parathyroid Tumors and Hormone Excess

A major clinical challenge in parathyroid disease involves cases associated with atypical features. The distinction between atypical parathyroid tumors and parathyroid carcinomas is critically important. In such cases, close communication of details between clinician and pathologist is exemplary of interdisciplinary care. Pathologists need to be aware of clinical details including serum calcium, vitamin D and renal status, intraoperative findings and parathyroid hormone level changes, glandular size and weight, and radionuclide and other imaging modalities; a critical clinical detail is the history of a previous biopsy or procedure in the neck [25, 26]. Similarly, clinicians seek details in the pathological report that are definitional for parathyroid carcinoma and include according to the 2022 WHO classification: angioinvasion (vascular invasion), lymphatic invasion, perineural (or intraneural) invasion, local malignant invasion into adjacent anatomic structures, or histologically/cytologically documented metastatic disease [10].

#### Gastroenteropancreatic (GEP) NETs and Hormone Excess

In GEP tumors, the detection of unexpected hormone expression, such as somatostatin, may drive its investigation when not suspected clinically. Alternatively, the absence of serotonin reactivity in a NET can divert surveillance with the cumbersome 24-h urinary 5-hydroxy indole acetic acid (5-HIAA), the breakdown product of serotonin, as in this case, these measurements have no bearing to the tumor in question. For example, a pancreatic, appendiceal, or rectal NET that expresses pancreatic polypeptide but not serotonin would not lend itself to 5-HIAA surveillance. At the other end of the spectrum are situations where the hormone elevation drives the search for the occult endocrine neoplasm. A prismatic scenario is ectopic Cushing syndrome where the source of ACTH is sought in an occult pancreatic NEN. Multiple and secondary hormone secretion can be found in 3–10% of patients with metastatic pancreatic neoplasms. This may occur at diagnosis (e.g., a gastrinoma co-secreting ACTH) or may be metachronous (i.e., may develop over time). Secondary hormone secretion is usually associated with disease progression and is also associated with increased morbidity and mortality, particularly in patients with newly diagnosed insulin hypersecretion, but also in patients with ectopic ACTH and PTHrP production [27–30].

A 56-year-old male was diagnosed with a clinically non-functioning grade 2 TxN1M1 neuroendocrine tumor of the pancreatic tail with lymph node and multilobar liver metastases. He was treated with a long-acting somatostatin analog. He developed progressive muscle weakness, particularly of the upper legs, requiring treatment for hypertension and edema, and blood tests showed severe hypokalemia, and blood glucose levels were two-fold elevated over the upper limits of normal (ULN). Further analysis showed that the patient had a tenfold elevated urinary cortisol excretion, and ACTH levels in the blood were fivefold elevated over the ULN. The patient was diagnosed with ectopic Cushing syndrome caused by ACTH secretion by the metastatic pancreatic NEN, was treated with cortisol-lowering drugs, and underwent an endoscopic bilateral adrenalectomy to control his severe hypercortisolism. A biopsy of a liver metastasis was taken during adrenal surgery, and immunohistochemistry for ACTH was positive. This staining was negative on the initial biopsy of the tumor and of a liver metastasis at diagnosis.

In an era of the common use of proton pump inhibitors (PPIs) and upper gastrointestinal endoscopies, hypergastrinemia has become a frequent clinical encounter. Many patients undergo endoscopic biopsies where the pathologist is confronted with a diagnostic dilemma. The distinction between the indolent type I gastric NET from the potentially more serious forms of gastric NENs becomes crucial. In addition to the close examination of features of autoimmune atrophic gastritis, the importance of clinical history and biochemical findings is increasingly obvious. The conditions under which the serum gastrin and chromogranin levels were obtained, prior use of PPIs, and the presence/absence of inappropriately elevated gastric pH become essential information in case assessment.

### Pathological and Imaging Correlates in Endocrine Pathology

**Fig. 2:**
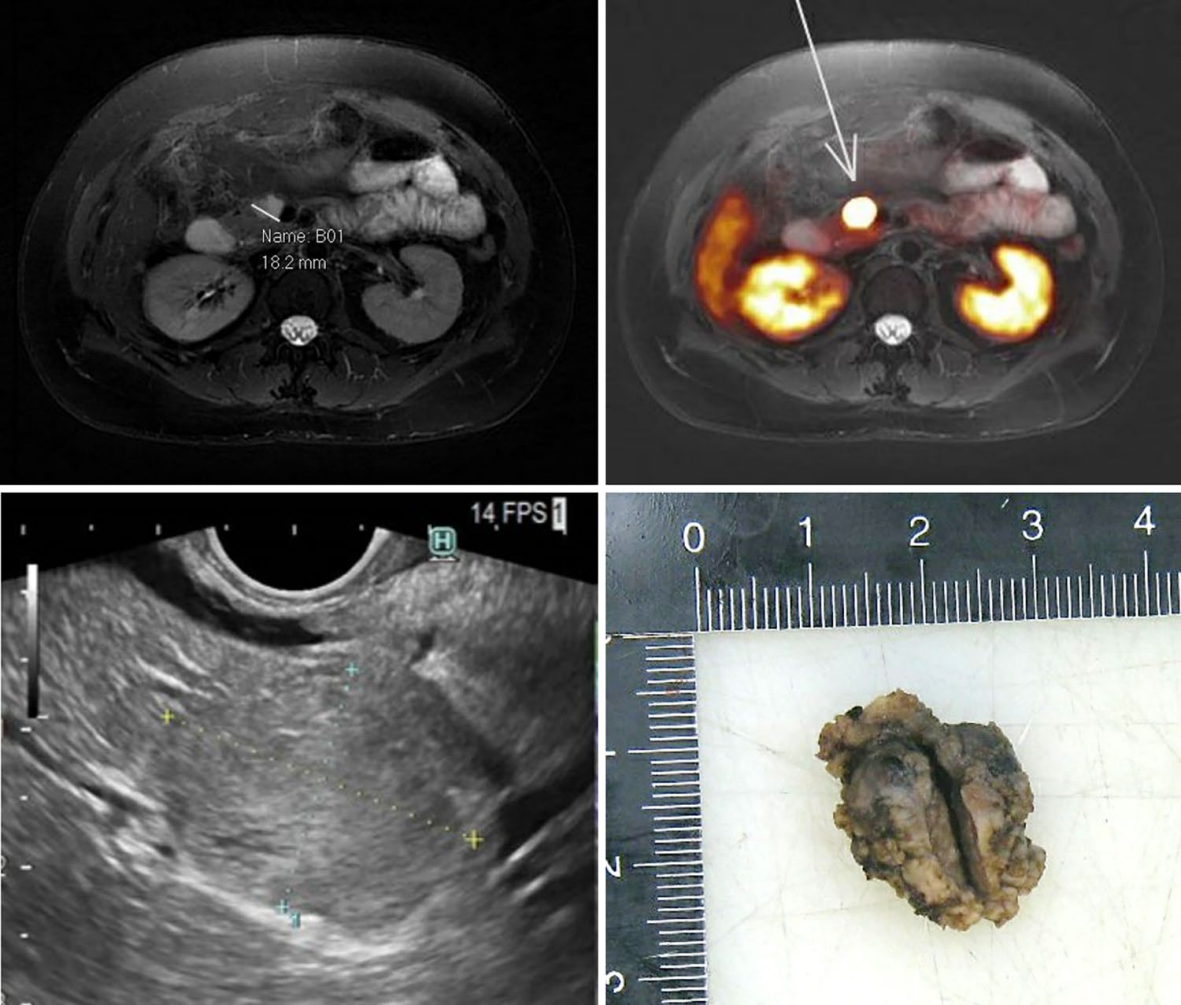
43-year-old woman with hyperinsulinemic hypoglycemia caused by an SSTR2-positive Grade 1 T1 insulin-producing pancreatic NET with a diameter of 1 cm. Upper panels, from left to right: MRI (T2W) shows a high T2 intense hypervascular lesion in the region of the pancreatic head which is positive on the ^68^Ga-DOTATATE PET MRI. Lower panels, from left to right: endoscopic ultrasound shows an isoechoic lesion that was resected endoscopically

Along with biochemical studies, structural and functional imaging provide the second pillar for the basis of clinical decision-making. In some instances, the imaging findings are fairly straightforward, with a single target matching the clinical impression (Fig. 2). However, in many other instances, the imaging studies yield more findings than initially anticipated. We identify below some clinical scenarios where endocrine pathology provides pivotal information essential for rational decision-making.

#### Chest Imaging and Lung Neuroendocrine Tumors

**Fig. 3:**
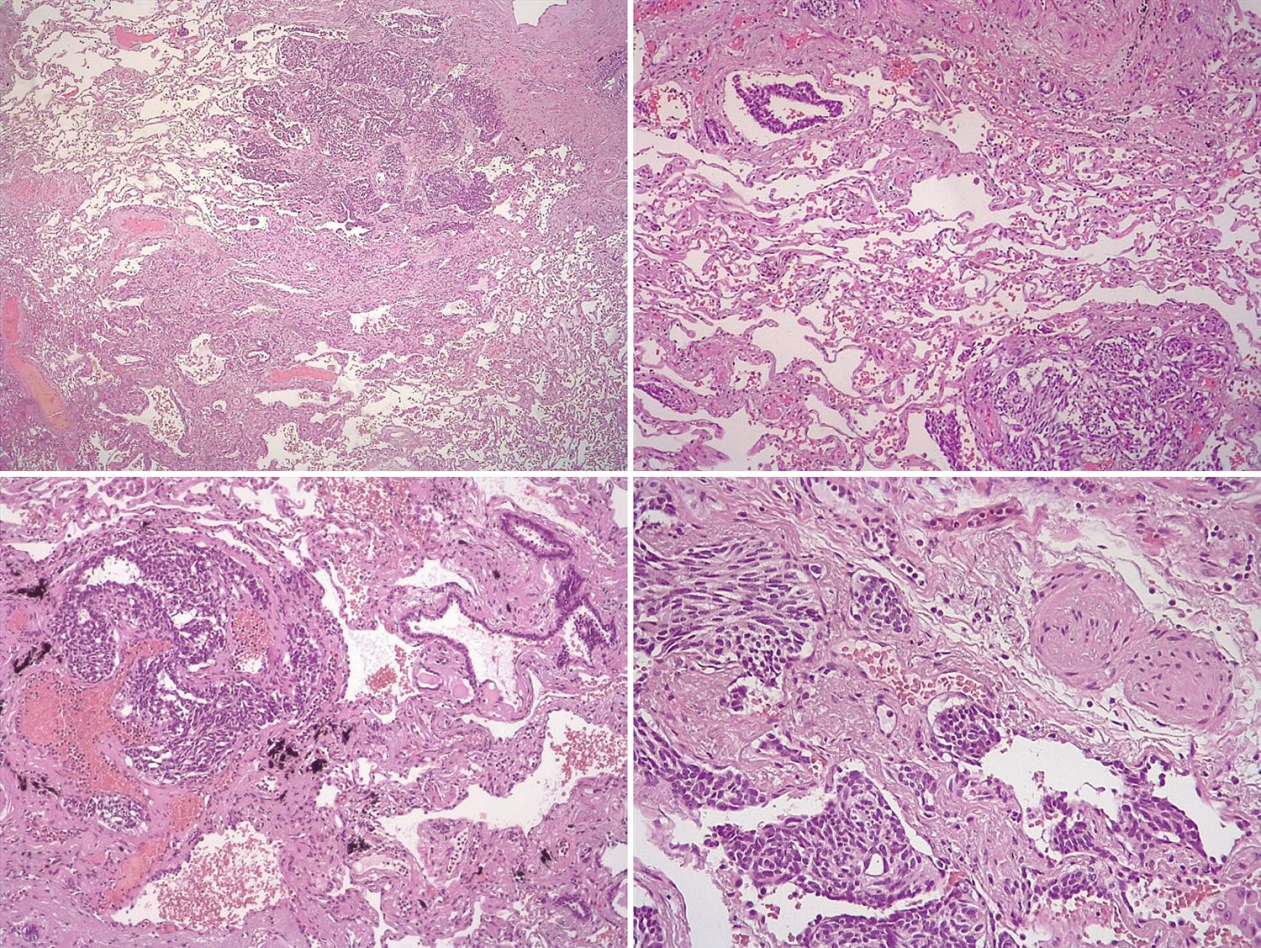
Diffuse Idiopathic Pulmonary Neuroendocrine Cell Hyperplasia (DiPNECH). A case of a female patient, aged 62 years, with multiple (#3) well-differentiated pulmonary neuroendocrine tumors (typical carcinoids) (not shown in the figure) associated with multiple pulmonary neuroendocrine microtumors (tumorlets) on a background of interstitial fibrosis. The patient had a former clinical diagnosis of asthma

An obese (BMI of 38 kg/m^2^) 55-year-old female was seen by a respiratory physician with a problem of mild dyspnea and was diagnosed with asthma. However, “routine” chest radiology revealed a 2 cm lung nodule and at least 4 further nodules approximately 5 mm in diameter. Endobronchial ultrasound (EBUS) and biopsy showed the large nodule to be a NET. A ^68^Ga-DOTATATE PET CT demonstrated intense uptake in the nodule, but the smaller lesions were thought to be below the limit of resolution. Video-assisted thoracoscopic surgery (VATS) resulted in an R0 resection of a low-grade/Grade 1 well-differentiated NET (“typical carcinoid”), and the patient was reassured that the long-term outlook was excellent. However, further review by an expert histopathologist revealed that in addition, there were areas of neuroendocrine cell hyperplasia compatible with a diagnosis of DIPNECH (Diffuse Idiopathic Pulmonary Neuroendocrine Cell Hyperplasia). Despite being based on still-debated criteria, the 5th edition of the World Health Organization classification of pulmonary neuroendocrine tumors introduced essential and desirable diagnostic criteria for the clinical and pathologic diagnoses of DIPNECH, which currently represents one entity in a spectrum of disorders rather than a unique disease [31] (Fig. 3). Controversies still exist on the clinical impact of NETs in the context of DIPNECH, but it is recommended that in such cases, the follow-up and clinical management should be tailored based on a multidisciplinary discussion [32]. Literature data suggest that multifocal pulmonary neuroendocrine proliferations, including pulmonary neuroendocrine microtumors (tumorlets) and pulmonary neuroendocrine cell hyperplasia, represent a clinically and prognostic relevant factor in well-differentiated pulmonary neuroendocrine tumors (carcinoid tumors), being associated with a higher risk of lymph node spread and of tumor relapse [33]. In the patient described, the prognosis was therefore more guarded, as these patients have a measurable risk of long-term metastatic disease [34], and close follow-up was initiated.

A 38-year-old female presented with a short history of florid Cushing’s syndrome, confirmed biochemically with a plasma ACTH of 355 ng/L. Pituitary MRI was essentially normal, while CT scanning revealed a 1 cm subcarinal node of uncertain significance. A ^68^Ga-DOTATATE PET CT showed positivity in this node but none elsewhere; EBUS and biopsy revealed a grade 1 (Ki-67 < 2%) NET, and a VATS lobectomy led to a biochemical and clinical cure. Routine follow-up over several years has shown no recurrence.

#### Abdominal Imaging and Retroperitoneal Lesions

The distinction between multifocal primaries versus metastatic disease is particularly important. This requires the integration of clinical, genetic, and biochemical studies with imaging findings. A common example is noted in patients with pathogenic germline *SDHx* mutations, where multiple paragangliomas in the retroperitoneum need to be distinguished from metastatic disease in lymph nodes. Radiographically, lymph nodes and retroperitoneal paragangliomas are almost indistinguishable as they assume nearly identical anatomical locations [35]. The detection of paragangliomas in tissues outside of where their normal counterparts normally reside (i.e., lymph nodes or bone) is required for confirmation of metastatic disease. In this regard, functional imaging such as ^68^Ga-DOTATATE PET/CT can be helpful. This now standard approach to detect somatostatin receptor subtypes using molecular functional imaging not only helps identify occult disease, but will also reveal skeletal metastases not otherwise identified. Furthermore, histopathologic examination must take into account the clinical, biochemical, and imaging findings. Needless to say, the ultimate clinical decision of commencing post-operative systemic therapy versus continued surveillance hinges on the validity of this information. Tumoral somatostatin receptor subtype 2a (SSTR2a) expression using immunohistochemistry correlates with in vivo somatostatin receptor imaging and with responsiveness to peptide receptor radionuclide therapy (PRRT) using radiolabeled somatostatin analogs. The combination of the same compound (or a very similar one) used for diagnostic imaging and therapy is the basis for the term “theranostics.” However, current PRRT protocols require the demonstration of sufficient uptake on in vivo somatostatin receptor imaging, usually performed using ^68^Ga-DOTA labeled somatostatin analogs [36]. Multifocality in paragangliomas and small bowel NETs can also be demonstrated using ^68^Ga-DOTA labeled somatostatin analog PET [37, 38].

#### Pelvic Imaging and Endocrine Tumors

##### Adrenal Rest Tissue and Tumors

This is a clinical situation where adrenal tissue is detected outside of the normal retroperitoneal sites of residence. The most typical area is the broad ligament resected as part of a hysterectomy specimen. However, adrenal rests occur within the testis and ovary, kidney and liver, and other pelvic locations including in prostatectomy or cystectomy specimens [39] as well as other more rare sites (WHO). The critical question for the clinician is to distinguish benign adrenal rest tissue from (i) secondary proliferations of ectopic adrenal tissue, (ii) hyperplasia to adenoma transitions, (iii) Leydig cell tumors, (iv) steroidogenic gonadal tumors, or even (v) metastatic adrenocortical carcinoma. In this context, SF1 expression may be misleading in the differential diagnosis with other steroidogenic tissues and tumors, such as steroidogenic gonadal neoplasms (e.g., steroid cell tumor, Leydig cell tumor). Adrenal cortex-specific steroidogenic enzymes, such as CYP11B1 and CYP21A2, may provide additional value to define primary adrenal cortical origin in extra-adrenal locations [11].

Again, knowledge of the patient’s clinical background, genetic predisposition such as congenital enzymatic deficiencies, endocrine status, and prior therapies is crucial in resolving this diagnostic puzzle. Nevertheless, the clinical implications of such a differential diagnosis justify such efforts.

**Fig. 4:**
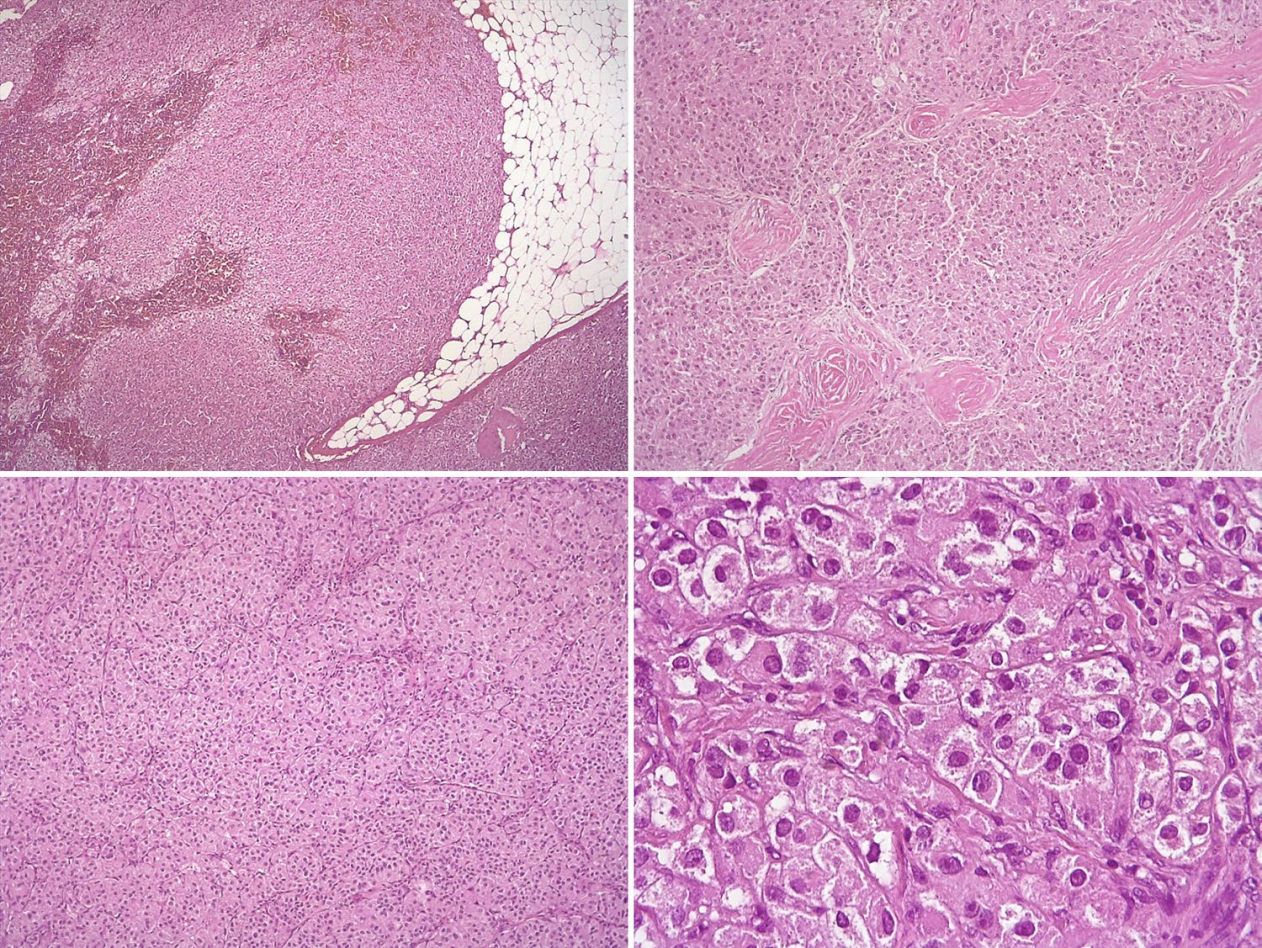
Worrisome morphological features that may be misinterpreted as signs of malignancy in adrenocortical tumors. Upper panel: a case of a female patient, aged 73 years, with an adrenal lesion 7 cm in largest dimension associated with hypercortisolemia. The lesion was diagnosed as adrenocortical carcinoma based on pseudo-invasion of the tumor capsule (left, myelo-lipomatous areas also present) and the presence of fibrous bands (right), but no other Weiss parameters were present. Lower panels: a case of a female patient, aged 35 years, with an adrenal lesion 2.5 cm in largest dimension, non-secretory. The lesion was diagnosed as adrenocortical carcinoma based on diffuse growth and eosinophilic cytoplasm (left) and the presence of nuclear atypia with sparse prominent nucleoli (right), but the overall findings (including a Ki-67 of < 1%) were consistent with a diagnosis of adrenocortical adenoma, oncocytic variant

**Fig. 5:**
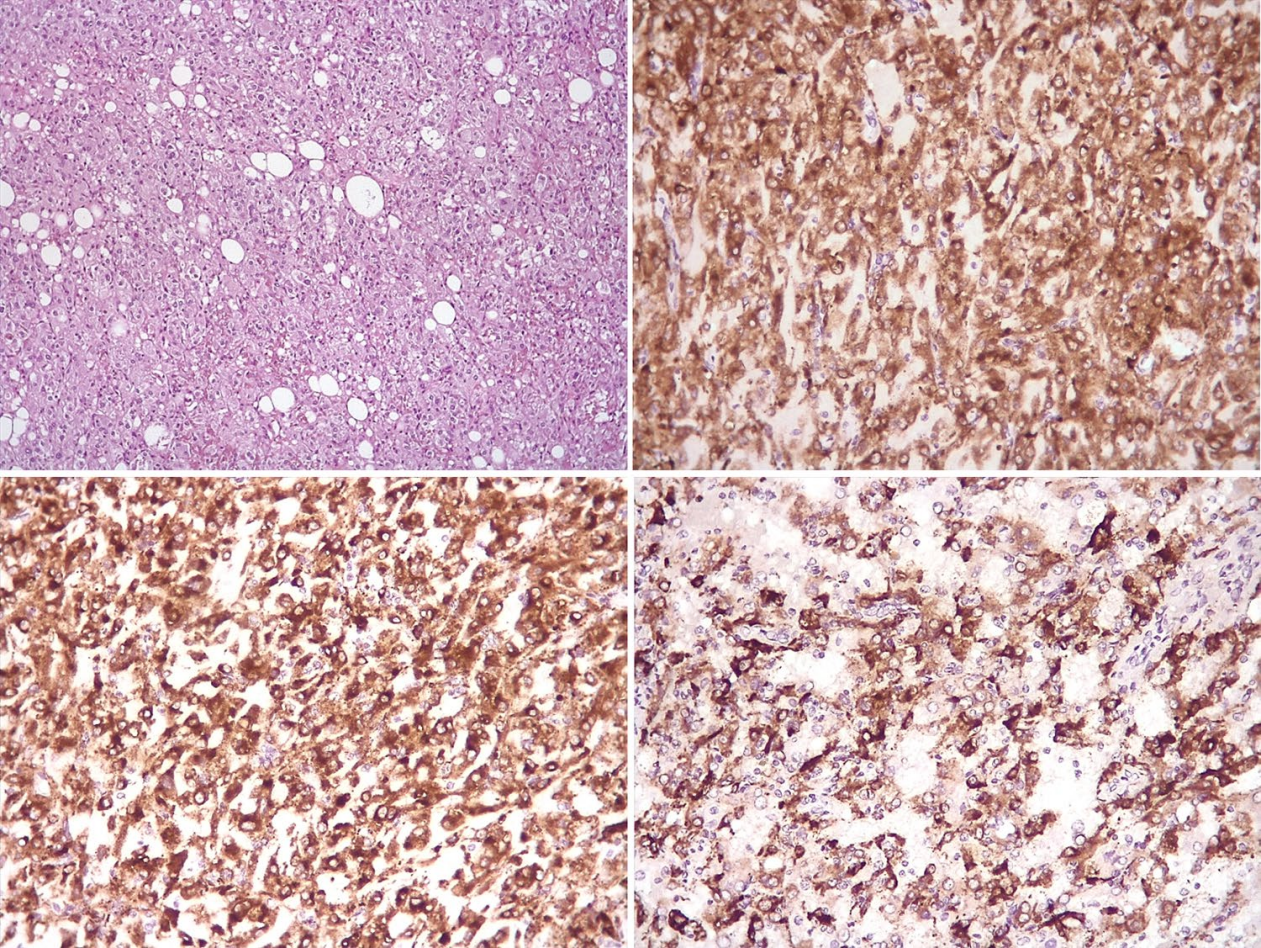
Adrenal lesion misdiagnosed as an adrenocortical carcinoma. A case of a female patient, aged 52 years, with an adrenal lesion 4.6 cm in largest dimension, clinically non-functioning. The epithelioid appearance was associated with intense and diffuse staining for Melan A (top right), but SF-1 was negative, whereas both cathepsin K (bottom left) and HMB45 (bottom right) were positive leading to a diagnosis of a perivascular epithelioid cell tumor (PEComa)

The information sought in adrenal endocrine pathology reporting range from the more benign to the malignant spectrum of adrenal disorders. Biopsy sampling is often quite limited in its ability to provide complete information which must await surgical pathology. The diagnosis of malignancy in adrenal cortical neoplasms can be complex, and diagnostic pitfalls may include, among others, either the misinterpretation of malignancy-related pathological findings or the erroneous attribution of adrenocortical origin to morphological mimickers of adrenocortical carcinoma (Figs. 4 and 5). In the hyperaldosteronism setting, CYP11B2 immunohistochemistry can assist in the detection of functional sites associated with bilateral disease [40]. Similarly, examination of the unaffected adrenal tissue for atrophic changes can alert clinicians to the presence of unrecognized subclinical Cushing’s syndrome (“autonomous cortisol secretion”).

At the other end of the spectrum, but a critical area in clinical decision-making, is the risk stratification of adrenal carcinomas. Identifying the hallmarks of adrenocortical cancer (ACC) that contribute to the various scoring systems such as the Weiss and Helsinki systems requires transparent communication between pathologist and clinician [41]. Importantly, clinicians need to understand the extent to which features such as vascular (angio-) invasion have been critically assessed by the pathologist. Similarly, tumor proliferation rates based on the mitotic count and Ki-67 labeling index are sought carefully in interpreting the basis for adrenocortical carcinoma risk stratification. This information has a direct impact on commencing adjuvant therapies, particularly when post-operative staging studies are unremarkable.

##### Teratomas and Their Tumors

The pelvis is a frequent site of germ cell-derived tumors with composite tissue elements, referred to as teratomas. Cystic teratomas can give rise to neoplasms of three main groups: (i) neuroendocrine tumors, sometimes referred to as “ovarian carcinoids” [42], (ii) the even more familiar thyroid tissue proliferations known as “struma ovarii” that may have thyroid cancer elements, or (iii) mixed struma/NENs. Recognizing this clinical spectrum helps align the clinician with the pathologist. Within this spectrum, rare NEN types may also occur such as primary paragangliomas or PitNETs, both functional and non-functional, including rare cases of mixed sparsely granulated lactotroph and densely granulated somatotroph tumors [43].

Confronted with the new diagnosis of NEN in the pelvis, abdomen, or chest, this requires close examination of a much older specimen as this may have been part of a remote oophorectomy. A careful clinical history can often help the pathologist, underscoring the need for clinic-pathological correlation. In the setting of malignant struma ovarii diagnosis, the clinician needs to risk-stratify the patient to determine the need for adjuvant therapy including possible thyroidectomy and radio-iodine administration [44]. The mere description of size without clarification of the extent of solid, cystic, and malignant elements is not satisfactory. Again, the potential impact of clinical interventions, particularly in a pre-menopausal female, highlights the importance of detailed histopathologic assessments.

##### Appendiceal NETs

The extent to which appendiceal NETs can be regarded as incidental findings versus clinically relevant neoplasms is an increasingly common dilemma. The following example represents such a scenario. A 14-year-old boy developed acute appendicitis and had an emergency appendectomy. The pathologist identified an inflamed appendix, as expected, as well as an appendiceal: he was scheduled for an urgent hemicolectomy. The parents became very concerned that he had “cancer and demanded a second opinion. At a *Centre of Excellence* for neuroendocrine tumors, the expert histopathologist diagnosed a 12 mm NET at the tip of the appendix, immunostaining for pan-cytokeratins, synaptophysin, and chromogranin A, and showing L-cell differentiation with expression of glucagon and pancreatic polypeptide; the tumor was negative for serotonin. The Ki-67 was 1%, and there was no lymphovascular or meso-appendiceal invasion. The parents were reassured and informed that according to recent studies, right hemicolectomy was not required [45–47].

## Pathological and Genetic Correlates in Endocrine Pathology

Unique morphologic features of an endocrine tumor can also instruct clinicians to initiate investigations for a specific genetic alteration. Here, we will provide a short list of such examples in different clinical settings.

### Pituitary Tumors

A 25-year-old patient complains of carpal tunnel syndrome; after a year of seeing various specialists, the diagnosis of acromegaly is suspected, and she is eventually referred to an endocrinologist. She has an elevated age- and sex-related serum insulin-like growth factor 1 (IGF-1). She fails to suppress growth hormone (GH) in a glucose tolerance test and is confirmed to have a large pituitary tumor on MRI. Trans-sphenoidal surgery provides a histopathological specimen which shows a tumor immunostaining for GH but is sparsely granulated with fibrous bodies on keratin staining. This in turn leads the endocrinologist to question the patient in more detail and discovers at least two other family members with pituitary tumors. Genetic testing reveals a germline *AIP* mutation, while the granulation pattern also suggests that the patient will be poorly responsive to first-generation somatostatin analogs (Octreotide/Lanreotide) for any residual excess GH secretion, although she may respond better to the second-generation pasireotide.

Hereditary predisposition is recognized in about 5% of PitNETs and may appear as either isolated familial PitNETs or syndromic tumors [48]. Somatotroph and/or mammosomatroph hyperplasia can signal other germline conditions including McCune-Albright and Carneys’ complex [49]. Another example is the identification of extensive cytoplasmic vacuolation in a pituitary tumor suggesting the presence of a germline *SDHx* mutation [50].

### Thyroid Carcinoma

In thyroid pathology, the presence of distinctive morphological findings should alert the pathologist to a possible familial cancer syndrome. For example, the recognition of a cribriform-morular thyroid carcinoma should prompt the clinician to investigate a familial adenomatous polyposis (FAP) pathogenesis. Similarly, a work-up for PTEN-hamartoma tumor syndrome should be considered in the presence of multiple adenomatous nodules in the thyroid of a young patient [51].

### Medullary Thyroid Cancer

Due to the generally low incidence of medullary thyroid cancer (MTC) among thyroid lesions, it is not unusual that the diagnosis is first established by the pathologist. In this context, the detection of multifocality and/or C-cell hyperplasia is important not only for the diagnosis of MTC, but also for defining potential germline-associated disease such as familial MTC that occurs in multiple endocrine neoplasia type 2A (MEN2A) or MEN2B [52]. Again, the critical assessment of proliferative and invasive features provides essential information in guiding management and surveillance plans. The recent development of an international medullary thyroid carcinoma grading system addresses some of these issues [53].

### Gastroenteropancreatic (GEP) Tumors

The detection of a keratin-negative pancreatic, duodenal, or other neuroendocrine tumor should prompt investigation for a possible paraganglioma that warrants investigation for the possible genetic predispositions of these tumors. In other instances, the clinician may have overlooked the possibility of a heritable etiology when faced with uncovering of multifocal endocrine tumors or precursor lesions, as in MEN, von Hippel Lindau (VHL), or *SDH*-mutated syndromes. Precursor lesions of GEP tumors that are suggestive of inherited conditions include gastric enterochromaffin-like cell (ECL) hyperplasia and gastrin-producing and/or somatostatin-producing cell hyperplasia that are associated to MEN1-related type II gastric and duodenal NETs, respectively, or islet hyperplasia, islet dysplasia and ductulo-insular complexes in the setting of MEN1- or VHL-related pancreatic NETs [54, 55].

### Cancers with Neuroendocrine Features

Cancer patients, most typically those treated for breast or prostate adenocarcinoma, routinely undergo imaging surveillance. In some instances, the presence of germline genetic predisposition may render this patient at risk of endocrine in addition to non-endocrine carcinomas. Regardless, cancer surveillance settings can lead to the encounter of a biopsy diagnosis of neuroendocrine disease which can be confusing and challenging. The clinician is confronted with decisions that require clearer distinctions. In scenario “A,” the recurrence of the adenocarcinoma is confounded by the presence of occasional neuroendocrine cells or focal neuroendocrine differentiation. Interpretation of biopsy findings, including classical immunohistochemistry for markers of neuroendocrine differentiation, requires a detailed knowledge of the clinical background and imaging findings. In this context, the neuroendocrine features become more of a “distraction,” perhaps inappropriately leading the clinician down the path of neuroendocrine disease investigation and management. This is to be distinguished from scenario “B” of a true NEN. In the “C” scenario, the tumor is a true mixed NEN and non-NEN, currently denoted as (MiNEN) [56]. In this latter context of MiNEN, each of the components of the disease may require a distinct management plan. This can include a sequential approach where the non-NEN component is prioritized for expedited management with chemotherapy. Depending on the clinical outcome, the NEN component can then follow in the usual fashion.

According to the 2022 WHO classification, MiNENs are neoplasms in which the two components are malignant, are morphologically and immunohistochemically recognizable, and each of them represents at least 30% of the tumor burden [57]. They are clearly distinguishable from non-neuroendocrine carcinomas (adenocarcinomas, urothelial carcinomas, squamous cell carcinomas, or others) with interspersed neuroendocrine cells since these latter are only identifiable using immunohistochemistry but are not morphologically recognizable. More problematic is the differential diagnosis with amphicrine carcinomas, which can be found in both digestive and extra-digestive sites. They represent “hybrid neoplasms” showing morphological, immunohistochemical, and ultrastructural features of both neuroendocrine and non-neuroendocrine differentiation in the same cells, but lack the typical organoid and neuroendocrine-like morphology and co-express both neuroendocrine and non-neuroendocrine markers in the same cells [58].

## The Pathologist at the Patient’s Bedside

Apart from “remote” diagnostic activity examining pathology specimens, pathologists may play a dynamic role by collaborating with clinicians at the bedside of the patient, with “point of contact” diagnosis. The two main situations for a synchronous intervention of pathologists in patients’ clinical decision-making are represented by intraoperative consultation and the Rapid On-Site Evaluation (ROSE) for the control of the quality and quantity of cells obtained in fine needle aspiration cytology (FNAC). The latter is especially helpful for patients whose anxiety may be alleviated by discussion of the findings on-site rather than having to await a further consultation.

### Thyroid Carcinomas

Intraoperative assessment of tissue samples is focused on answering clinical questions that will drive further intraoperative surgical procedures. Although limited by the suboptimal quality of slides obtained from frozen tissues (as compared to formalin-fixed and paraffin-embedded material), in general, intraoperative frozen section examination yields a high concordance with the final diagnosis (97%) on permanent sections and by a low number of cases with deferred diagnosis (3.1%) [59]. In thyroid pathology, it is less valuable, particularly in the assessment of follicular nodules where it has a low predictive value [60]; however, it may be used in patients with papillary thyroid carcinoma to assess cervical lymph node (level II–V) metastasis, although FNAB cytology and thyroglobulin FNAB needle wash-testing have been shown to have a superior sensitivity [61]. Moreover, it is useful to identify parathyroid tissue and to decrease post-op hypoparathyroidism in patients undergoing total thyroidectomy.

Much has been discussed about the benefits of ROSE in recent years, in different fields of pathology.

In thyroid cytology, several studies have indicated a significant decrease in non-diagnostic findings in the presence of ROSE, as compared to the absence of ROSE, in a percentage of around 20% in different series [62–64]. Therefore, ROSE is currently considered as the standard of care for thyroid FNABs. Application of ROSE also reduces the mean number of FNABs per nodule [62], reducing the risk of complications for the patient.

### Parathyroid Tumors

In parathyroid pathology, with special reference to primary hyperparathyroidism, intraoperative frozen section examination of all removed tissues corresponds well with pre-operative imaging techniques (such as 4D-CT), and in combination with intraoperative assessment of parathyroid hormone levels represents the best approach to detect pathological parathyroid tissue, a decrease in surgical complication rates, and an improvement in outcomes [65].

### Pituitary Tumors

In pituitary pathology, frozen section examination is of unique value in situations where the differential diagnosis includes metastatic or inflammatory conditions. While in Cushing disease it may be helpful [66], caution must be exercised not to waste the already minimal tissue on frozen studies. In this setting, permanent sections must be preserved for morphologic identification not only of the corticotroph tumor but the Crooke’s hyaline changes. In some instances, it is only the latter ancillary features that provide support for the accuracy of the diagnosis of true Cushing.

### Gastroenteropancreatic (GEP) NENs

In neuroendocrine tumors, diagnostic cytology is particularly relevant in endoscopic ultrasound-guided fine needle aspiration cytology (EUS-FNA) of pancreatic and intra-abdominal lesions. The impact of ROSE in improving diagnostic accuracy is not as established as it is in thyroid cytology, although ROSE has shown a high concordance rate (98.2%) with the final cyto-pathological diagnosis in some series [67].

In GEP neuroendocrine neoplasms, the role of intraoperative frozen section examination is less well established and varies greatly according to the location of the tumor. The main reasons for performing frozen sections are the identification of the lesion, assessment of the extent of disease, and the evaluation of the adequacy of resection [68].

## The Pathologist as a Core Member of Multidisciplinary Team

Pathologists are members of the multidisciplinary care team, and their role is essential to integrate the patient care process in both the pre-operative and the post-operative settings.

Endocrine pathology has become more and more complex in the last decades due to our deeper understanding of endocrine tumors in their different aspects, from pathogenesis, to pathways of progression, to a widening of therapeutic strategies. This also led, apart from refining the diagnostic approaches, to the definition of biomarkers of prognostic and predictive value whose evaluation needs consolidated and robust methodologies as well as a correct integration into the patient’s clinical management, leading to a clinical meaningful interpretation. Aspects related to the application of immunophenotyping and molecular pathology in endocrine tumors are discussed in detail in other papers; however, we emphasize that almost all endocrine tumors have a significant proportion of inherited/familial cases, and awareness of a high incidence of familial disease is a core issue for a multidisciplinary discussion.

The overall complexity of this field justifies the need for a dedicated subspecialty that bridges pathology with endocrinology and endocrine oncology. However, a significant number of patients endure a series of encounters that delay diagnosis and therapy due to incomplete or incorrect information. If this holds true for different fields of oncology patients, it is specifically an issue for patients affected by endocrine tumors whose management may overlap with a wide variety of disciplines. As an example, adrenocortical carcinoma integrates endocrinology for the evaluation and management of hormonal manifestations, oncology for chemo/immunotherapy, radiotherapy for loco-regional treatments, urology for surgical management, and genetics for the recognition of inherited disease [69]. In neuroendocrine tumors, with special reference to those of the GEP system, together with endocrinologists, oncologists, radiotherapists, surgeons, and geneticists as mentioned above, the ideal team will also include gastroenterologists, nuclear medicine specialists, nutritionists, and nurse specialists. Thus, while the best way that all these disciplines can be involved is in a multidisciplinary meeting (MDT), this need is frequently unmet, and patients may often be managed based on the specialization of the first clinician to whom the patient presents. Irrespective of the main clinical presentation, pathologists are able to provide information potentially useful for all these clinical colleagues and therefore are central in the process. In fact, it is the pathologist’s responsibility to interpret pathological findings in the correct scenario (i.e., surgical versus pre-surgical setting, histological versus cytological samples, first diagnostic setting versus disease progression or tumor location) with specific adherence to the “clinical question.”

The limiting factor in the development of subspecialty pathology has been the number of pathologists required in any organization to support every discipline. New technologies such as digital pathology are allowing a new model of pathology to emerge, enabling the consolidation of pathologists into large groups supporting multi-site institutions and virtual consultation of complete diagnostic material for second opinions [70]. Moreover, the digital pathology approach allows the potential integration of the pathology diagnosis and clinical information through the application of artificial intelligence tools, such as machine learning, a field of current research of promising applicability in different fields of endocrine oncology [71].

Not to be forgotten is the fact that pathologists are also trained physicians. With current advances in electronic patient records, a new era of communication has emerged. Patients are increasingly interested in seeking to communicate directly with those involved in their diagnoses. Pathologists will likely play an increasing role in communicating directly with patients. The unique complexities of endocrine pathology highlight this need and predict another dimension to the multidisciplinary care of patients.

## Tissue Biobanking: A Pathologist’s Responsibility

Biobanking of high-quality material, combined with clinical and imaging data, is a fundamental tool to facilitate research and can and should contribute to the discovery of clinically relevant biological markers for optimal decision-making [72, 73]. This covers potentially any aspect of endocrine pathology, with special reference to endocrine oncology, and represents a milestone in integrating diagnostic and research pathology in a translational approach. However, the application of tissue biobanking procedures has to take into account several aspects that cannot be solved unilaterally by pathologists or clinicians acting in isolation, but needs to be approached in a multidisciplinary environment.

The main issues relate to the fact that (i) the request by scientists for human samples with proven biological quality and multilayered sets of annotations (including molecular signatures) is constantly increasing, (ii) biobanking procedures have to fully meet the criteria of safety of the personnel working with biological products, and (iii) laws and regulations that integrate the ethical and societal dimension of biobanking are constantly under modification and are subject to varying jurisdictional regulations.

A clear example of the impact on research of prospective biobanking procedures in endocrine pathology is represented by the *Chernobyl Tissue Bank* that was established after the nuclear accident in Chernobyl in 1986. The project was built by the governments of Ukraine and Russia and financially supported by the *European Commission*, the *National Cancer Institute* of the USA, and the *Sasakawa Memorial Health Foundation* of Japan. Tissues collected starting from 1988 supplied material to 21 research projects in Japan, the USA, and Europe, providing a paradigm for cancer research in the molecular biological age [74].

Other more recent examples in endocrine oncology have been reported in Europe (i.e., at the Pasteur Hospital, Nice, France) and Canada with the aim of developing translational research projects on thousands of cases [70, 75–78]. They emphasize the need for standardized procedures to obtain high-quality biological resources and clinical annotations. Indeed, as the number of international networks for research programs using biological products is steadily increasing, it is crucial to achieve harmonization on biobanking procedures. Even the size and weight of tissue fragments are not typically coded in a standardized approach, although they may influence the adequacy of biological material for planned analyses [79].

Moreover, the aims of tissue biobanking are currently not limited to the availability of representative tumor tissue fragments, but also embed the establishment of functional and/or in vivo tumor models, such as primary cell cultures, organoids, or patient-derived xenografts. Primary cell cultures obtained from adrenocortical carcinoma patients have been demonstrated to be useful tools to determine profiles of responsiveness to mitotane [80]. In the neuroendocrine field, the potential of tumor organoids as a model for GEP neuroendocrine carcinoma has been tested to assess chemosensitivity in parallel with the patient’s clinical response [81, 82]. Similar work on pheochromocytoma and paraganglioma human organoids has explored the possibility that such in vitro models, in combination with next-generation sequencing, may identify optimal drugs and their combinations, which are likely to be therapeutically effective [83].

A special issue is related to the storage and banking of biopsies or cytological material. This is particularly relevant in the clinics for thyroid fine needle aspiration (FNA) biopsy material. In fact, one of the most crucial aspects of thyroid cytology is the quality of the samples. The failure to obtain high-quality samples not only may lead to inaccurate diagnoses, but poor quality specimens may be also not be suitable for biobanking, thus failing to provide useful biological information at the time of diagnosis or prospectively. In this respect, ROSE is a helpful tool not only for the assessment of sampling adequacy, but also in the triage of the sample allowing to obtain adequate material for further ancillary techniques. Long-term storage of thyroid FNA cytological samples at − 80 °C has proved to guarantee high-quality nucleic acid material [84], but it is not easy to establish in all laboratories, since it needs space and equipment, and stored material may lack any kind of morphological evaluation. Therefore, biobanking might better rely on standard procedures to process cytological material, which have been developed to incorporate microscopic evaluation with immunohistochemical and molecular techniques. Apart from smears, the liquid-based cytology approach and cell block preparations are tools to store cytological material for additional analyses concurrent with or following morphological evaluation [85]. As for tissue specimens, the applicability of ancillary methods on cytological specimens is dependent on the availability of high-quality samples. Accordingly, there is a growing need to harmonize pre-analytical steps, from technical aspects of sampling procedures to optimization of long-term storage/biobanking methods.

Finally, requirements related to ethical and legal issues have to be considered. A proper definition of biobanks includes large collections of bio-specimens linked to relevant personal and health information (health records, family history, lifestyle, genetic information) that are held predominantly for use in health and medical research. In principle, the *International Organization for Standardization* states in the requirements for biobanking (ISO 20387:2018; ISO (2018) International Standard ISO 20387:2018—Biotechnology—Biobanking—General requirements for biobanking, First Edit. https://www.iso.org/standard/67888.html) that biobanks are legal entities driving the process of acquisition and storage together with some or all of the activities related to collection, preparation, preservation, testing, analyzing, and distributing defined biological material, as well as related information and data. Thus, biobanks should follow strict rules in terms of safety, reliability, and efficiency designed in international infrastructures to facilitate networking, encourage education, improve standardization, and support recognition of biobanks as a vital part of scientific productivity [86]. They should also include robust consent procedures such that patients are at all times aware of the value and use of these samples and, where relevant, are party to any commercial considerations. Throughout these efforts, it is important to ensure that research biobanks do not compromise clinically essential diagnostic tissue [87].

## Data Availability

Not applicable.

## References

[1] Gruppen LD, Woolliscroft JO, Wolf FM (1988). The contribution of different components of the clinical encounter in generating and eliminating diagnostic hypotheses. Res Med Educ.

[2] Srigley JR, Judge M, Helliwell T, Birdsong GG, Ellis DW (2021) The International Collaboration on Cancer Reporting (ICCR): a decade of progress towards global pathology standardisation and data interoperability. Histopathology 79:897–901. 10.1111/his.14431. 10.1111/his.14431 34783048

[3] Ghossein R, Barletta JA, Bullock M, Johnson SJ, Kakudo K, Lam AK, Moonim MT, Poller DN, Tallini G, Tuttle RM, Xu B, Gill AJ (2021) Data set for reporting carcinoma of the thyroid: recommendations from the International Collaboration on Cancer Reporting. Hum Pathol 110:62–72. 10.1016/j.humpath.2020.08.009. 10.1016/j.humpath.2020.08.009 PMC7943644 32920035

[4] Williams MD, DeLellis RA, Erickson LA, Gupta R, Johnson SJ, Kameyama K, Natu S, Ng T, Perren A, Perrier ND, Seethala RR, Gill AJ (2021) Pathology data set for reporting parathyroid carcinoma and atypical parathyroid neoplasm: recommendations from the International Collaboration on Cancer Reporting. Hum Pathol 110:73–82. 10.1016/j.humpath.2020.07.008. 10.1016/j.humpath.2020.07.008 32687943

[5] Giordano TJ, Berney D, de Krijger RR, Erickson L, Fassnacht M, Mete O, Papathomas T, Papotti M, Sasano H, Thompson LDR, Volante M, Gill AJ (2021) Data set for reporting of carcinoma of the adrenal cortex: explanations and recommendations of the guidelines from the International Collaboration on Cancer Reporting. Hum Pathol 110:50–61. 10.1016/j.humpath.2020.10.001. 10.1016/j.humpath.2020.10.001 33058949

[6] Thompson LDR, Gill AJ, Asa SL, Clifton-Bligh RJ, de Krijger RR, Kimura N, Komminoth P, Lack EE, Lenders JWM, Lloyd RV, Papathomas TG, Sadow PM, Tischler AS (2021) Data set for the reporting of pheochromocytoma and paraganglioma: explanations and recommendations of the guidelines from the International Collaboration on Cancer Reporting. Hum Pathol 110:83–97. 10.1016/j.humpath.2020.04.012. 10.1016/j.humpath.2020.04.012 PMC7655677 32407815

[7] van Velthuysen MF, Couvelard A, Rindi G, Fazio N, Hörsch D, Nieveen van Dijkum EJ, Klöppel G, Perren A (2022) ENETS standardized (synoptic) reporting for neuroendocrine tumour pathology. J Neuroendocrinol 34:e13100. 10.1111/jne.13100. 10.1111/jne.13100 PMC9285411 35165954

[8] Nosé V, Ezzat S, Horvath E, Kovacs K, Laws ER, Lloyd R, Lopes MB, Asa SL (2011) Protocol for the examination of specimens from patients with primary pituitary tumors. Arch Pathol Lab Med 135:640–6. 10.5858/2010-0470-SAR1.1. 10.5858/2010-0470-SAR1.1 21526962

[9] Baloch ZW, Asa SL, Barletta JA, Ghossein RA, Juhlin CC, Jung CK, LiVolsi VA, Papotti MG, Sobrinho-Simões M, Tallini G, Mete O (2022) Overview of the 2022 WHO Classification of Thyroid Neoplasms. Endocr Pathol 33:27–63. 10.1007/s12022-022-09707-3. 10.1007/s12022-022-09707-3 35288841

[10] Erickson LA, Mete O, Juhlin CC, Perren A, Gill AJ (2022) Overview of the 2022 WHO Classification of Parathyroid Tumors. Endocr Pathol 33:64–89. 10.1007/s12022-022-09709-1. 10.1007/s12022-022-09709-1 35175514

[11] Mete O, Erickson LA, Juhlin CC, de Krijger RR, Sasano H, Volante M, Papotti MG (2022) Overview of the 2022 WHO Classification of Adrenal Cortical Tumors. Endocr Pathol 33:155–196. 10.1007/s12022-022-09710-8. 10.1007/s12022-022-09710-8 PMC8920443 35288842

[12] Mete O, Asa SL, Gill AJ, Kimura N, de Krijger RR, Tischler A (2022) Overview of the 2022 WHO Classification of Paragangliomas and Pheochromocytomas. Endocr Pathol 33:90–114. 10.1007/s12022-022-09704-6. 10.1007/s12022-022-09704-6 35285002

[13] Cibas ES, Ali SZ (2017) The 2017 Bethesda System for Reporting Thyroid Cytopathology. Thyroid 27:1341–1346. 10.1089/thy.2017.0500. 10.1089/thy.2017.0500 29091573

[14] Tomaszewski JE, Bear HD, Connally JA, Epstein JI, Feldman M, Foucar K, Layfield L, LiVolsi V, Sirota RL, Stoler MH, Stombler RE (2000) Consensus conference on second opinions in diagnostic anatomic pathology. Who, What, and When. Am J Clin Pathol 114:329–335. 10.1093/ajcp/114.3.329. 10.1093/ajcp/114.3.329 10989631

[15] Simpson PR, Tschang TP (1993) ADASP recommendations: consultations in surgical pathology. Association of Directors of Anatomic and Surgical Pathology. Hum Pathol 24:1382. 10.1016/0046-8177(93)90276-m. 10.1016/0046-8177(93)90276-m 8276389

[16] Epstein JI, Walsh PC, Sanfilippo F (1996) Clinical and cost impact of second-opinion pathology. Review of prostate biopsies prior to radical prostatectomy. Am J Surg Pathol 20:851–857. 10.1097/00000478-199607000-00008. 10.1097/00000478-199607000-00008 8669533

[17] Farooq A, Abdelkader A, Javakhishivili N, Moreno GA, Kuderer P, Polley M, Hunt B, Giorgadze TA, Jorns JM (2021) Assessing the value of second opinion pathology review. Int J Qual Health Care 33:mzab032. 10.1093/intqhc/mzab032. 10.1093/intqhc/mzab032 33644816

[18] Duregon E, Volante M, Bollito E, Goia M, Buttigliero C, Zaggia B, Berruti A, Scagliotti GV, Papotti M (2015) Pitfalls in the diagnosis of adrenocortical tumors: a lesson from 300 consultation cases. Hum Pathol 46:1799–1807. 10.1016/j.humpath.2015.08.012. 10.1016/j.humpath.2015.08.012 26472162

[19] Merola E, Zandee W, de Mestier L, Klümpen HJ, Makulik K, Geboes K, van Velthuysen ML, Couvelard A, Cros J, van Eeden S, Hoorens A, Stephenson T, Zajęcki W, de Herder W, Munir A (2021) Histopathological Revision for Gastroenteropancreatic Neuroendocrine Neoplasms in Expert Centers: Does It Make the Difference? Neuroendocrinology 111:170–177. 10.1159/000507082. 10.1159/000507082 32155627

[20] Swarts DR, van Suylen RJ, den Bakker MA, van Oosterhout MF, Thunnissen FB, Volante M, Dingemans AM, Scheltinga MR, Bootsma GP, Pouwels HM, van den Borne BE, Ramaekers FC, Speel EJ (2014) Interobserver variability for the WHO classification of pulmonary carcinoids. Am J Surg Pathol 38:1429–1436. 10.1097/PAS.0000000000000300. 10.1097/PAS.0000000000000300 25046341

[21] Cree IA (2022) From Counting Mitoses to Ki67 Assessment: Technical Pitfalls in the New WHO Classification of Endocrine and Neuroendocrine Tumors. Endocr Pathol 33:3–5. 10.1007/s12022-021-09701-1. 10.1007/s12022-021-09701-1 35028827

[22] La Rosa S (2023) Diagnostic, Prognostic, and Predictive Role of Ki67 Proliferative Index in Neuroendocrine and Endocrine Neoplasms: Past, Present, and Future. Endocr Pathol 34:79–97. 10.1007/s12022-023-09755-3. 10.1007/s12022-023-09755-3 PMC10011307 36797453

[23] Luchini C, Pantanowitz L, Adsay V, Asa SL, Antonini P, Girolami I, Veronese N, Nottegar A, Cingarlini S, Landoni L, Brosens LA, Verschuur AV, Mattiolo P, Pea A, Mafficini A, Milella M, Niazi MK, Gurcan MN, Eccher A, Cree IA, Scarpa A (2022) Ki-67 assessment of pancreatic neuroendocrine neoplasms: Systematic review and meta-analysis of manual vs. digital pathology scoring. Mod Pathol 35:712–720. 10.1038/s41379-022-01055-1. 10.1038/s41379-022-01055-1 PMC9174054 35249100

[24] Potorac I, Bonneville JF, Daly AF, de Herder W, Fainstein-Day P, Chanson P, Korbonits M, Cordido F, Baranski Lamback E, Abid M, Raverot V, Raverot G, Anda Apiñániz E, Caron P, Du Boullay H, Bildingmaier M, Bolanowski M, Laloi-Michelin M, Borson-Chazot F, Chabre O, Christin-Maitre S, Briet C, Diaz-Soto G, Bonneville F, Castinetti F, Gadelha MR, Oliveira Santana N, Stelmachowska-Banaś M, Gudbjartsson T, Villar-Taibo R, Zornitzki T, Tshibanda L, Petrossians P, Beckers A (2022) Pituitary MRI Features in Acromegaly Resulting From Ectopic GHRH Secretion From a Neuroendocrine Tumor: Analysis of 30 Cases. J Clin Endocrinol Metab 107:e3313-e3320. 10.1210/clinem/dgac274. 10.1210/clinem/dgac274 35512251

[25] Akirov A, Asa SL, Larouche V, Mete O, Sawka AM, Jang R, Ezzat S (2019) The Clinicopathological Spectrum of Parathyroid Carcinoma. Front Endocrinol (Lausanne) 10:731. 10.3389/fendo.2019.00731. 10.3389/fendo.2019.00731 PMC6819433 31708875

[26] Roser P, Leca BM, Coelho C, Schulte KM, Gilbert J, Drakou EE, Kosmas C, Chuah LL, Wassati H, Miras AD, Crane J, Aylwin SJB, Grossman AB, Dimitriadis GK (2023) Diagnosis and management of parathyroid carcinoma: a state of the art review. Endocr Relat Cancer ERC-22–0287. 10.1530/ERC-22-0287. 10.1530/ERC-22-0287 36621911

[27] de Mestier L, Hentic O, Cros J, Walter T, Roquin G, Brixi H, Lombard-Bohas C, Hammel P, Diebold MD, Couvelard A, Ruszniewski P, Cadiot G (2015) Metachronous hormonal syndromes in patients with pancreatic neuroendocrine tumors: a case-series study. Ann Intern Med 162:682–689. 10.7326/M14-2132. 10.7326/M14-2132 25984844

[28] Crona J, Norlén O, Antonodimitrakis P, Welin S, Stålberg P, Eriksson B (2016) Multiple and Secondary Hormone Secretion in Patients With Metastatic Pancreatic Neuroendocrine Tumours. J Clin Endocrinol Metab 101:445–452. 10.1210/jc.2015-2436. 10.1210/jc.2015-2436 26672633

[29] Kamp K, Alwani RA, Korpershoek E, Franssen GJ, de Herder WW, Feelders RA (2016) Prevalence and clinical features of the ectopic ACTH syndrome in patients with gastroenteropancreatic and thoracic neuroendocrine tumors. Eur J Endocrinol 174:271–280. 10.1530/EJE-15-0968. 10.1530/EJE-15-0968 26643855

[30] Kamp K, Feelders RA, van Adrichem RC, de Rijke YB, van Nederveen FH, Kwekkeboom DJ, de Herder WW (2014) Parathyroid hormone-related peptide (PTHrP) secretion by gastroenteropancreatic neuroendocrine tumors (GEP-NETs): clinical features, diagnosis, management, and follow-up. J Clin Endocrinol Metab 99:3060–3069. 10.1210/jc.2014-1315. 10.1210/jc.2014-1315 24905065

[31] Tassi V, Daddi N, Mete O (2023) Not All Multifocal Pulmonary Neuroendocrine Cell Proliferations Represent Diffuse Idiopathic Pulmonary Neuroendocrine Cell Hyperplasia. Ann Thorac Surg 115:547–548. 10.1016/j.athoracsur.2022.05.009. 10.1016/j.athoracsur.2022.05.009 35597259

[32] Ramirez RA, Cass AS, Das S, Low SW, Mehrad M, Rickman OB, Scherer PM, Thomas KE, Gillaspie EA (2022) A multidisciplinary approach to the work up and management of pulmonary carcinoid tumors and DIPNECH: a narrative review. Transl Lung Cancer Res 11:2567–2587. 10.21037/tlcr-22-415. 10.21037/tlcr-22-415 PMC9830261 36636417

[33] Tassi V, Scarnecchia E, Ferolla P, Mete O, Manjula M, Allison F, Potenza R, Vannucci J, Ceccarelli S, Yasufuku K, De Perrot M, Pierre A, Darling G, Colella R, Ascani S, Mattioli S, Keshavjee S, Waddell TK, Puma F, Daddi N (2022) Prognostic Significance of Pulmonary Multifocal Neuroendocrine Proliferation With Typical Carcinoid. Ann Thorac Surg 113:966–974. 10.1016/j.athoracsur.2021.03.069. 10.1016/j.athoracsur.2021.03.069 33831394

[34] Hayes AR, Luong TV, Banks J, Shah H, Watkins J, Lim E, Patel A, Grossman AB, Navalkissoor S, Krell D, Caplin ME (2022) Diffuse idiopathic pulmonary neuroendocrine cell hyperplasia (DIPNECH): Prevalence, clinicopathological characteristics and survival outcome in a cohort of 311 patients with well-differentiated lung neuroendocrine tumours. J Neuroendocrinol 34:e13184. 10.1111/jne.13184. 10.1111/jne.13184 36121922

[35] Asa SL, Ezzat S, Mete O (2018) The Diagnosis and Clinical Significance of Paragangliomas in Unusual Locations. J Clin Med 7:280. 10.3390/jcm7090280. 10.3390/jcm7090280 PMC6162705 30217041

[36] van Adrichem RC, Kamp K, van Deurzen CH, Biermann K, Feelders RA, Franssen GJ, Kwekkeboom DJ, Hofland LJ, de Herder WW (2016) Is There an Additional Value of Using Somatostatin Receptor Subtype 2a Immunohistochemistry Compared to Somatostatin Receptor Scintigraphy Uptake in Predicting Gastroenteropancreatic Neuroendocrine Tumor Response? Neuroendocrinology 103:560–566. 10.1159/000441604. 10.1159/000441604 26536001

[37] Norlén O, Montan H, Hellman P, Stålberg P, Sundin A (2018) Preoperative 68Ga-DOTA-Somatostatin Analog-PET/CT Hybrid Imaging Increases Detection Rate of Intra-abdominal Small Intestinal Neuroendocrine Tumor Lesions. World J Surg 42:498–505. 10.1007/s00268-017-4364-1. 10.1007/s00268-017-4364-1 PMC5762814 29159606

[38] Ambrosini V, Kunikowska J, Baudin E, Bodei L, Bouvier C, Capdevila J, Cremonesi M, de Herder WW, Dromain C, Falconi M, Fani M, Fanti S, Hicks RJ, Kabasakal L, Kaltsas G, Lewington V, Minozzi S, Cinquini M, Öberg K, Oyen WJG, O'Toole D, Pavel M, Ruszniewski P, Scarpa A, Strosberg J, Sundin A, Taïeb D, Virgolini I, Wild D, Herrmann K, Yao J (2021) Consensus on molecular imaging and theranostics in neuroendocrine neoplasms. Eur J Cancer 146:56–73. 10.1016/j.ejca.2021.01.008. 10.1016/j.ejca.2021.01.008 PMC8903070 33588146

[39] Falco EC, Daniele L, Metovic J, Bollito E, De Rosa G, Volante M, Papotti M (2021) Adrenal Rests in the Uro-genital Tract of an Adult Population. Endocr Pathol 32:375–384. 10.1007/s12022-021-09685-y. 10.1007/s12022-021-09685-y PMC8370964 34095993

[40] Nakamura Y, Maekawa T, Felizola SJ, Satoh F, Qi X, Velarde-Miranda C, Plonczynski MW, Ise K, Kikuchi K, Rainey WE, Gomez-Sanchez EP, Gomez-Sanchez CE, Sasano H (2014) Adrenal CYP11B1/2 expression in primary aldosteronism: immunohistochemical analysis using novel monoclonal antibodies. Mol Cell Endocrinol 392:73–79. 10.1016/j.mce.2014.05.002. 10.1016/j.mce.2014.05.002 PMC5471353 24837548

[41] Duregon E, Cappellesso R, Maffeis V, Zaggia B, Ventura L, Berruti A, Terzolo M, Fassina A, Volante M, Papotti M (2017) Validation of the prognostic role of the "Helsinki Score" in 225 cases of adrenocortical carcinoma. Hum Pathol 62:1–7. 10.1016/j.humpath.2016.09.035. 10.1016/j.humpath.2016.09.035 27916625

[42] Opalińska M, Sowa-Staszczak A, Olearska H, Ulatowska-Bialas M, Gilis-Januszewska A, Hubalewska-Dydejczyk A (2021) Clinical Approach to Neuroendocrine Neoplasm Associated With Ovarian Teratoma. Front Endocrinol (Lausanne) 12:770266. 10.3389/fendo.2021.770266. 10.3389/fendo.2021.770266 PMC8670552 34917031

[43] Hodgson A, Pakbaz S, Shenouda C, Francis JA, Mete O (2020) Mixed Sparsely Granulated Lactotroph and Densely Granulated Somatotroph Pituitary Neuroendocrine Tumor Expands the Spectrum of Neuroendocrine Neoplasms in Ovarian Teratomas: the Role of Pituitary Neuroendocrine Cell Lineage Biomarkers. Endocr Pathol 31:315–319. 10.1007/s12022-020-09639-w. 10.1007/s12022-020-09639-w 32632841

[44] Janszen EW, van Doorn HC, Ewing PC, de Krijger RR, de Wilt JH, Kam BL, de Herder WW (2008) Malignant struma ovarii: good response after thyroidectomy and I ablation therapy. Clin Med Oncol 2:147–152. 10.4137/cmo.s410. 10.4137/cmo.s410 PMC3161682 21892277

[45] Nesti C, Bräutigam K, Benavent M, Bernal L, Boharoon H, Botling J, Bouroumeau A, Brcic I, Brunner M, Cadiot G, Camara M, Christ E, Clerici T, Clift AK, Clouston H, Cobianchi L, Ćwikła JB, Daskalakis K, Frilling A, Garcia-Carbonero R, Grozinsky-Glasberg S, Hernando J, Hervieu V, Hofland J, Holmager P, Inzani F, Jann H, Jimenez-Fonseca P, Kaçmaz E, Kaemmerer D, Kaltsas G, Klimacek B, Knigge U, Kolasińska-Ćwikła A, Kolb W, Kos-Kudła B, Kunze CA, Landolfi S, La Rosa S, López CL, Lorenz K, Matter M, Mazal P, Mestre-Alagarda C, Del Burgo PM, van Dijkum EJMN, Oleinikov K, Orci LA, Panzuto F, Pavel M, Perrier M, Reims HM, Rindi G, Rinke A, Rinzivillo M, Sagaert X, Satiroglu I, Selberherr A, Siebenhüner AR, Tesselaar MET, Thalhammer MJ, Thiis-Evensen E, Toumpanakis C, Vandamme T, van den Berg JG, Vanoli A, van Velthuysen MF, Verslype C, Vorburger SA, Lugli A, Ramage J, Zwahlen M, Perren A, Kaderli RM (2023) Hemicolectomy versus appendectomy for patients with appendiceal neuroendocrine tumours 1–2 cm in size: a retrospective, Europe-wide, pooled cohort study. Lancet Oncol 24:187–194. 10.1016/S1470-2045(22)00750-1. 10.1016/S1470-2045(22)00750-1 36640790

[46] Toumpanakis C, Fazio N, Tiensuu Janson E, Hörsch D, Pascher A, Reed N, O Apos Toole D, Nieveen van Dijkum E, Partelli S, Rinke A, Kos-Kudla B, Costa F, Pape UF, Grozinsky-Glasberg S, Scoazec JY; The ENETS 2016 Munich Advisory Board Participants; ENETS 2016 Munich Advisory Board Participants (2019) Unmet Needs in Appendiceal Neuroendocrine Neoplasms. Neuroendocrinology 108:37–44. 10.1159/000493894. 10.1159/000493894 30235454

[47] Pape UF, Niederle B, Costa F, Gross D, Kelestimur F, Kianmanesh R, Knigge U, Öberg K, Pavel M, Perren A, Toumpanakis C, O'Connor J, Krenning E, Reed N, O'Toole D; Vienna Consensus Conference participants (2016) ENETS Consensus Guidelines for Neuroendocrine Neoplasms of the Appendix (Excluding Goblet Cell Carcinomas). Neuroendocrinology 103:144–52. 10.1159/000443165. 10.1159/000443165 26730583

[48] Armeni E, Grossman A (2023) The Spectrum of Familial Pituitary Neuroendocrine Tumors. Endocr Pathol 34:57–78. 10.1007/s12022-022-09742-0. 10.1007/s12022-022-09742-0 36401106

[49] Asa SL, Ezzat S (2021) An Update on Pituitary Neuroendocrine Tumors Leading to Acromegaly and Gigantism. J Clin Med 10:2254. 10.3390/jcm10112254. 10.3390/jcm10112254 PMC8196981 34067494

[50] Dénes J, Swords F, Rattenberry E, Stals K, Owens M, Cranston T, Xekouki P, Moran L, Kumar A, Wassif C, Fersht N, Baldeweg SE, Morris D, Lightman S, Agha A, Rees A, Grieve J, Powell M, Boguszewski CL, Dutta P, Thakker RV, Srirangalingam U, Thompson CJ, Druce M, Higham C, Davis J, Eeles R, Stevenson M, O'Sullivan B, Taniere P, Skordilis K, Gabrovska P, Barlier A, Webb SM, Aulinas A, Drake WM, Bevan JS, Preda C, Dalantaeva N, Ribeiro-Oliveira A Jr, Garcia IT, Yordanova G, Iotova V, Evanson J, Grossman AB, Trouillas J, Ellard S, Stratakis CA, Maher ER, Roncaroli F, Korbonits M (2015) Heterogeneous genetic background of the association of pheochromocytoma/paraganglioma and pituitary adenoma: results from a large patient cohort. J Clin Endocrinol Metab 100:E531–541. 10.1210/jc.2014-3399. 10.1210/jc.2014-3399 PMC4333031 25494863

[51] Nosé V, Gill A, Teijeiro JMC, Perren A, Erickson L (2022) Overview of the 2022 WHO Classification of Familial Endocrine Tumor Syndromes. Endocr Pathol 33:197–227. 10.1007/s12022-022-09705-5. 10.1007/s12022-022-09705-5 35285003

[52] Larouche V, Akirov A, Thomas CM, Krzyzanowska MK, Ezzat S (2019) A primer on the genetics of medullary thyroid cancer. Curr Oncol 26:389–394. 10.3747/co.26.5553. 10.3747/co.26.5553 PMC6927790 31896937

[53] Xu B, Fuchs TL, Ahmadi S, Alghamdi M, Alzumaili B, Bani MA, Baudin E, Chou A, De Leo A, Fagin JA, Ganly I, Glover A, Hartl D, Kanaan C, Khneisser P, Najdawi F, Nigam A, Papachristos A, Repaci A, Spanheimer PM, Solaroli E, Untch BR, Barletta JA, Tallini G, Al Ghuzlan A, Gill AJ, Ghossein RA (2022) International Medullary Thyroid Carcinoma Grading System: A Validated Grading System for Medullary Thyroid Carcinoma. J Clin Oncol 40:96–104. 10.1200/JCO.21.01329. 10.1200/JCO.21.01329 PMC8683221 34731032

[54] Mete O, Tischler AS, de Krijger R, McNicol AM, Eisenhofer G, Pacak K, Ezzat S, Asa SL (2014) Protocol for the examination of specimens from patients with pheochromocytomas and extra-adrenal paragangliomas. Arch Pathol Lab Med 138:182–188. 10.5858/arpa.2012-0551-OA. 10.5858/arpa.2012-0551-OA PMC3909881 24476517

[55] Mete O, Asa SL (2013) Precursor lesions of endocrine system neoplasms. Pathology 45:316–30. 10.1097/PAT.0b013e32835f45c5. 10.1097/PAT.0b013e32835f45c5 23478233

[56] La Rosa S, Sessa F, Uccella S (2016) Mixed Neuroendocrine-Nonneuroendocrine Neoplasms (MiNENs): Unifying the Concept of a Heterogeneous Group of Neoplasms. Endocr Pathol 27:284–311. 10.1007/s12022-016-9432-9. 10.1007/s12022-016-9432-9 27169712

[57] Rindi G, Mete O, Uccella S, Basturk O, La Rosa S, Brosens LAA, Ezzat S, de Herder WW, Klimstra DS, Papotti M, Asa SL (2022) Overview of the 2022 WHO Classification of Neuroendocrine Neoplasms. Endocr Pathol 33:115–154. 10.1007/s12022-022-09708-2. 10.1007/s12022-022-09708-2 35294740

[58] La Rosa S (2021) Challenges in High-grade Neuroendocrine Neoplasms and Mixed Neuroendocrine/Non-neuroendocrine Neoplasms. Endocr Pathol 32:245–257. 10.1007/s12022-021-09676-z. 10.1007/s12022-021-09676-z PMC8116295 33786701

[59] Mohamed A, Hassan MM, Zhong W, Kousar A, Takeda K, Donthi D, Rizvi A, Majeed M, Younes AI, Ali A, Sutton A, Murray G, Thayyil A, Fallon J, Geisinger K (2022) A Quantitative and Qualitative Assessment of Frozen Section Diagnosis Accuracy and Deferral Rate Across Organ Systems. Am J Clin Pathol 158:692–701. 10.1093/ajcp/aqac115. 10.1093/ajcp/aqac115 36197800

[60] Ye Q, Woo JS, Zhao Q, Wang P, Huang P, Chen L, Li X, Xu K, Yong Y, Sung-Eun Yang S, Rao J (2017) Fine-Needle Aspiration Versus Frozen Section in the Evaluation of Malignant Thyroid Nodules in Patients With the Diagnosis of Suspicious for Malignancy or Malignancy by Fine-Needle Aspiration. Arch Pathol Lab Med 141:684–689. 10.5858/arpa.2016-0305-OA. 10.5858/arpa.2016-0305-OA 28447904

[61] Du W, Fang Q, Dai L, Fan J (2022) Fine-needle aspiration biopsy versus frozen section examination in assessing cervical lymph node metastasis in primary clinically positive neck papillary thyroid carcinoma. Diagn Cytopathol 50:217–222. 10.1002/dc.24935. 10.1002/dc.24935 35103414

[62] Fawcett C, Eppenberger-Castori S, Zechmann S, Hanke J, Herzog M, Savic Prince S, Christ ER, Ebrahimi F (2022) Effects of Rapid On-Site Evaluation on Diagnostic Accuracy of Thyroid Fine-Needle Aspiration. Acta Cytol 66:371–378. 10.1159/000522662. 10.1159/000522662 PMC9501752 35512664

[63] Muri R, Trippel M, Borner U, Weidner S, Trepp R (2022) The Impact of Rapid On-Site Evaluation on the Quality and Diagnostic Value of Thyroid Nodule Fine-Needle Aspirations. Thyroid 32:667–674. 10.1089/thy.2021.0551. 10.1089/thy.2021.0551 35236111

[64] Pastorello RG, Destefani C, Pinto PH, Credidio CH, Reis RX, Rodrigues TA, Toledo MC, De Brot L, Costa FA, do Nascimento AG, Pinto CAL, Saieg MA (2018) The impact of rapid on-site evaluation on thyroid fine-needle aspiration biopsy: A 2-year cancer center institutional experience. Cancer Cytopathol 126:846–852. 10.1002/cncy.22051. 10.1002/cncy.22051 30317695

[65] Aydin H, Dural AC, Sahbaz NA, Karli M, Guzey D, Akarsu C, Ferahman S, Piskinpasa H, Yegul D, Sipahi M, Koyuncu A, Altinay S, Karabulut M (2021) Clinical adaptation of auxiliary methods and multidisciplinary approach to changing trends in parathyroid surgery. Medicine (Baltimore) 100:e27160. 10.1097/MD.0000000000027160. 10.1097/MD.0000000000027160 PMC8483855 34596115

[66] Qiao N, Swearingen B, Hedley-Whyte ET, Tritos NA (2019) The Utility of Intraoperative Cytological Smear and Frozen Section in the Surgical Management of Patients with Cushing's Disease due to Pituitary Microadenomas. Endocr Pathol 30:180–188. 10.1007/s12022-019-09582-5. 10.1007/s12022-019-09582-5 31228001

[67] Mehmood S, Jahan A, Loya A, Yusuf MA (2015) Onsite cytopathology evaluation and ancillary studies beneficial in EUS-FNA of pancreatic, mediastinal, intra-abdominal, and submucosal lesions. Diagn Cytopathol 43:278–286. 10.1002/dc.23207. 10.1002/dc.23207 25088987

[68] Couvelard A, Sauvanet A (2008) Gastroenteropancreatic neuroendocrine tumors: indications for and pitfalls of frozen section examination. Virchows Arch 453:441–448. 10.1007/s00428-008-0678-6. 10.1007/s00428-008-0678-6 18839209

[69] Puglisi S, Perotti P, Cosentini D, Roca E, Basile V, Berruti A, Terzolo M (2018) Decision-making for adrenocortical carcinoma: surgical, systemic, and endocrine management options. Expert Rev Anticancer Ther 18:1125–1133. 10.1080/14737140.2018.1510325. 10.1080/14737140.2018.1510325 30117750

[70] Volynskaya Z, Chow H, Evans A, Wolff A, Lagmay-Traya C, Asa SL (2018) Integrated Pathology Informatics Enables High-Quality Personalized and Precision Medicine: Digital Pathology and Beyond. Arch Pathol Lab Med 142:369–382. 10.5858/arpa.2017-0139-OA. 10.5858/arpa.2017-0139-OA 28849944

[71] Thomasian NM, Kamel IR, Bai HX (2022) Machine intelligence in non-invasive endocrine cancer diagnostics. Nat Rev Endocrino 18:81–95. 10.1038/s41574-021-00543-9. 10.1038/s41574-021-00543-9 PMC8576465 34754064

[72] Yu YY, Zhu ZG (2010) Significance of biological resource collection and tumor tissue bank creation. World J Gastrointest Oncol 2:5–8. 10.4251/wjgo.v2.i1.5. 10.4251/wjgo.v2.i1.5 PMC2999151 21160810

[73] Ladd AC, O'Sullivan-Mejia E, Lea T, Perry J, Dumur CI, Dragoescu E, Garrett CT, Powers CN (2011) Preservation of fine-needle aspiration specimens for future use in RNA-based molecular testing. Cancer Cytopathol 119:102–110. 10.1002/cncy.20130. 10.1002/cncy.20130 PMC4627600 21287691

[74] Thomas GA, Bethel JA, Galpine A, Mathieson W, Krznaric M, Unger K (2011) Integrating research on thyroid cancer after Chernobyl--the Chernobyl Tissue Bank. Clin Oncol (R Coll Radiol) 23:276–281. 10.1016/j.clon.2011.01.503. 10.1016/j.clon.2011.01.503 PMC3391565 21345659

[75] Lassalle S, Hofman V, Ilie M, Butori C, Bonnetaud C, Gaziello MC, Selva E, Gavric-Tanga V, Guevara N, Castillo L, Santini J, Chabannon C, Hofman P (2011) Setting up a Prospective Thyroid Biobank for Translational Research: Practical Approach of a Single Institution (2004–2009, Pasteur Hospital, Nice, France). Biopreserv Biobank 9:9–19. 10.1089/bio.2010.0024. 10.1089/bio.2010.0024 24850201

[76] Cancer Genome Atlas Research Network (2014) Integrated genomic characterization of papillary thyroid carcinoma. Cell 159:676–690. 10.1016/j.cell.2014.09.050. 10.1016/j.cell.2014.09.050 PMC4243044 25417114

[77] Zheng S, Cherniack AD, Dewal N, Moffitt RA, Danilova L, Murray BA, Lerario AM, Else T, Knijnenburg TA, Ciriello G, Kim S, Assie G, Morozova O, Akbani R, Shih J, Hoadley KA, Choueiri TK, Waldmann J, Mete O, Robertson AG, Wu HT, Raphael BJ, Shao L, Meyerson M, Demeure MJ, Beuschlein F, Gill AJ, Sidhu SB, Almeida MQ, Fragoso MCBV, Cope LM, Kebebew E, Habra MA, Whitsett TG, Bussey KJ, Rainey WE, Asa SL, Bertherat J, Fassnacht M, Wheeler DA; Cancer Genome Atlas Research Network; Hammer GD, Giordano TJ, Verhaak RGW (2016) Comprehensive Pan-Genomic Characterization of Adrenocortical Carcinoma. Cancer Cell 29:723–736. 10.1016/j.ccell.2016.04.002. 10.1016/j.ccell.2016.04.002 PMC4864952 27165744

[78] Fishbein L, Leshchiner I, Walter V, Danilova L, Robertson AG, Johnson AR, Lichtenberg TM, Murray BA, Ghayee HK, Else T, Ling S, Jefferys SR, de Cubas AA, Wenz B, Korpershoek E, Amelio AL, Makowski L, Rathmell WK, Gimenez-Roqueplo AP, Giordano TJ, Asa SL, Tischler AS; Cancer Genome Atlas Research Network; Pacak K, Nathanson KL, Wilkerson MD (2017) Comprehensive Molecular Characterization of Pheochromocytoma and Paraganglioma. Cancer Cell 31:181–193. 10.1016/j.ccell.2017.01.001. 10.1016/j.ccell.2017.01.001 PMC5643159 28162975

[79] Nohle DG, Mandt RL, Couce ME, Parwani AV, Ayers LW (2018) Acceptable Weight Ranges for Research Tissue Procurement and Biorepositories, 2015–2017. Biopreserv Biobank 16:463–466. 10.1089/bio.2018.0068. 10.1089/bio.2018.0068 PMC6308276 30379574

[80] van Koetsveld PM, Creemers SG, Dogan F, Franssen GJH, de Herder WW, Feelders RA, Hofland LJ (2020) The Efficacy of Mitotane in Human Primary Adrenocortical Carcinoma Cultures. J Clin Endocrinol Metab 105:407–417. 10.1210/clinem/dgz001. 10.1210/clinem/dgz001 PMC7006231 31586196

[81] Dijkstra KK, van den Berg JG, Weeber F, van de Haar J, Velds A, Kaing S, Peters DDGC, Eskens FALM, de Groot DA, Tesselaar MET, Voest EE (2021) Patient-Derived Organoid Models of Human Neuroendocrine Carcinoma. Front Endocrinol (Lausanne) 12:627819. 10.3389/fendo.2021.627819. 10.3389/fendo.2021.627819 PMC7991829 33776923

[82] Kawasaki K, Toshimitsu K, Matano M, Fujita M, Fujii M, Togasaki K, Ebisudani T, Shimokawa M, Takano A, Takahashi S, Ohta Y, Nanki K, Igarashi R, Ishimaru K, Ishida H, Sukawa Y, Sugimoto S, Saito Y, Maejima K, Sasagawa S, Lee H, Kim HG, Ha K, Hamamoto J, Fukunaga K, Maekawa A, Tanabe M, Ishihara S, Hamamoto Y, Yasuda H, Sekine S, Kudo A, Kitagawa Y, Kanai T, Nakagawa H, Sato T (2020) An Organoid Biobank of Neuroendocrine Neoplasms Enables Genotype-Phenotype Mapping. Cell 183:1420–1435.e21. 10.1016/j.cell.2020.10.023. 10.1016/j.cell.2020.10.023 33159857

[83] Wang K, Schütze I, Gulde S, Bechmann N, Richter S, Helm J, Lauseker M, Maurer J, Reul A, Spoettl G, Klink B, William D, Knösel T, Friemel J, Bihl M, Weber A, Fankhauser M, Schober L, Vetter D, Broglie Däppen M, Ziegler CG, Ullrich M, Pietzsch J, Bornstein SR, Lottspeich C, Kroiss M, Fassnacht M, Wenter VUJ, Ladurner R, Hantel C, Reincke M, Eisenhofer G, Grossman AB, Pacak K, Beuschlein F, Auernhammer CJ, Pellegata NS, Nölting S (2022) Personalized drug testing in human pheochromocytoma/paraganglioma primary cultures. Endocr Relat Cancer 29:285–306. 10.1530/ERC-21-0355. 10.1530/ERC-21-0355 35324454

[84] Mathieson W, Betsou F, Myshunina T, Pushkarev V, Pushkarev V, Shinkarkina A, Voskoboynyk L, Thomas GA (2016) The effect of long-term -80°C storage of thyroid biospecimens on RNA quality and ensuring fitness for purpose. J Clin Pathol 69:1105–1108. 10.1136/jclinpath-2016-203697. 10.1136/jclinpath-2016-203697 PMC5637094 27235537

[85] Bode-Lesniewska B, Cochand-Priollet B, Straccia P, Fadda G, Bongiovanni M (2019) Management of thyroid cytological material, preanalytical procedures and bio-banking. Cytopathology 30:7–16. 10.1111/cyt.12586. 10.1111/cyt.12586 29885011

[86] Annaratone L, De Palma G, Bonizzi G, Sapino A, Botti G, Berrino E, Mannelli C, Arcella P, Di Martino S, Steffan A, Daidone MG, Canzonieri V, Parodi B, Paradiso AV, Barberis M, Marchiò C; Alleanza Contro il Cancro (ACC) Pathology and Biobanking Working Group (2021) Basic principles of biobanking: from biological samples to precision medicine for patients. Virchows Arch 479:233–246. 10.1007/s00428-021-03151-0. 10.1007/s00428-021-03151-0 PMC8275637 34255145

[87] Cheung CC, Martin BR, Asa SL (2013) Defining diagnostic tissue in the era of personalized medicine. CMAJ 185:135–9. 10.1503/cmaj.1205 10.1503/cmaj.120565 PMC3563886 22825998

